# The context-dependent, combinatorial logic of BMP signaling

**DOI:** 10.1016/j.cels.2022.03.002

**Published:** 2022-04-13

**Authors:** Heidi Klumpe, Matthew A. Langley, James M. Linton, Christina J. Su, Yaron E. Antebi, Michael B. Elowitz

**Affiliations:** 1 Division of Biology and Bioengineering, California Institute of Technology, Pasadena, CA 91125, USA; 2 Division of Chemistry and Chemical Engineering, California Institute of Technology, Pasadena, CA 91125, USA; 3 Department of Molecular Genetics, Weizmann Institute of Science, Rehovot 76100, Israel; 4 Howard Hughes Medical Institute, Chevy Chase, MD 20815, USA; 5 Department of Applied Physics, California Institute of Technology, Pasadena, CA 91125, USA; 6 Lead Contact

**Keywords:** bone morphogenetic protein, BMP, signaling pathways, cell context, combinatorial signaling, promiscuous ligand-receptor interactions, pairwise interaction analysis

## Abstract

Cell-cell communication systems typically comprise families of ligand and receptor variants that function together in combinations. Pathway activation depends in a complex way on which ligands are present and what receptors are expressed by the signal-receiving cell. To understand the combinatorial logic of such a system, we systematically measured pairwise Bone Morphogenetic Protein (BMP) ligand interactions in cells with varying receptor expression. Ligands could be classified into equivalence groups based on their profile of positive and negative synergies with other ligands. These groups varied with receptor expression, explaining how ligands can functionally replace each other in one context but not another. Context-dependent combinatorial interactions could be explained by a biochemical model based on competitive formation of alternative signaling complexes with distinct activities. Together, these results provide insights into the roles of BMP combinations in developmental and therapeutic contexts and establish a framework for analyzing other combinatorial, context-dependent signaling systems.

## eTOC blurb

In BMP signaling, combinations of ligands interact with combinations of receptors to control diverse developmental processes. By measuring pathway responses to all pairs of ten BMP ligands, we classified the seemingly redundant BMP ligands into distinct contextual equivalence groups whose structure depends on the receptor expression profile of the target cells. The entire data set was consistent with a mathematical model of combinatorial signaling complex formation, and could explain the distinct roles of these homologous ligands in development.

## Graphical abstract

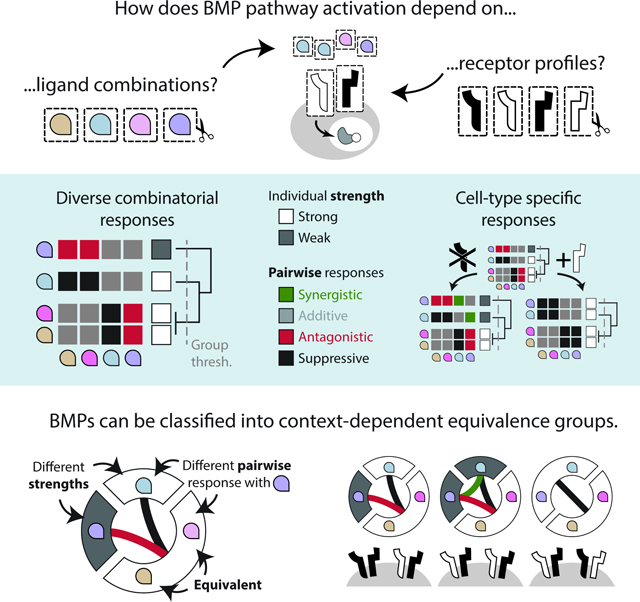

## Introduction

Cell-cell communication pathways such as BMP, Wnt, and FGF play pivotal roles in normal development, disease, therapeutics, and regenerative medicine (Nusse, 2005; Ornitz and Itoh, 2015; Salazar et al., 2016). A striking feature of these pathways is their use of families of homologous ligand variants. Within each pathway, multiple ligands typically function together in combinations (Alok et al., 2017; Antebi et al., 2017; Kadam et al., 2012). Further, the effect of any given ligand can vary dramatically with cell or tissue context (Chen et al., 2013; Eubelen et al., 2018; Fortin et al., 2005; Kadam et al., 2009; Verkaar and Zaman, 2010). Despite the prevalence of these features, and extensive information about the biochemical interactions and developmental roles of particular ligands, we lack a unified description of how ligands signal in combinations and across biological contexts. Understanding the principles of contextual combinatorial signaling could allow more predictive control of these pathways in natural and synthetic systems.

**Figure 1:**
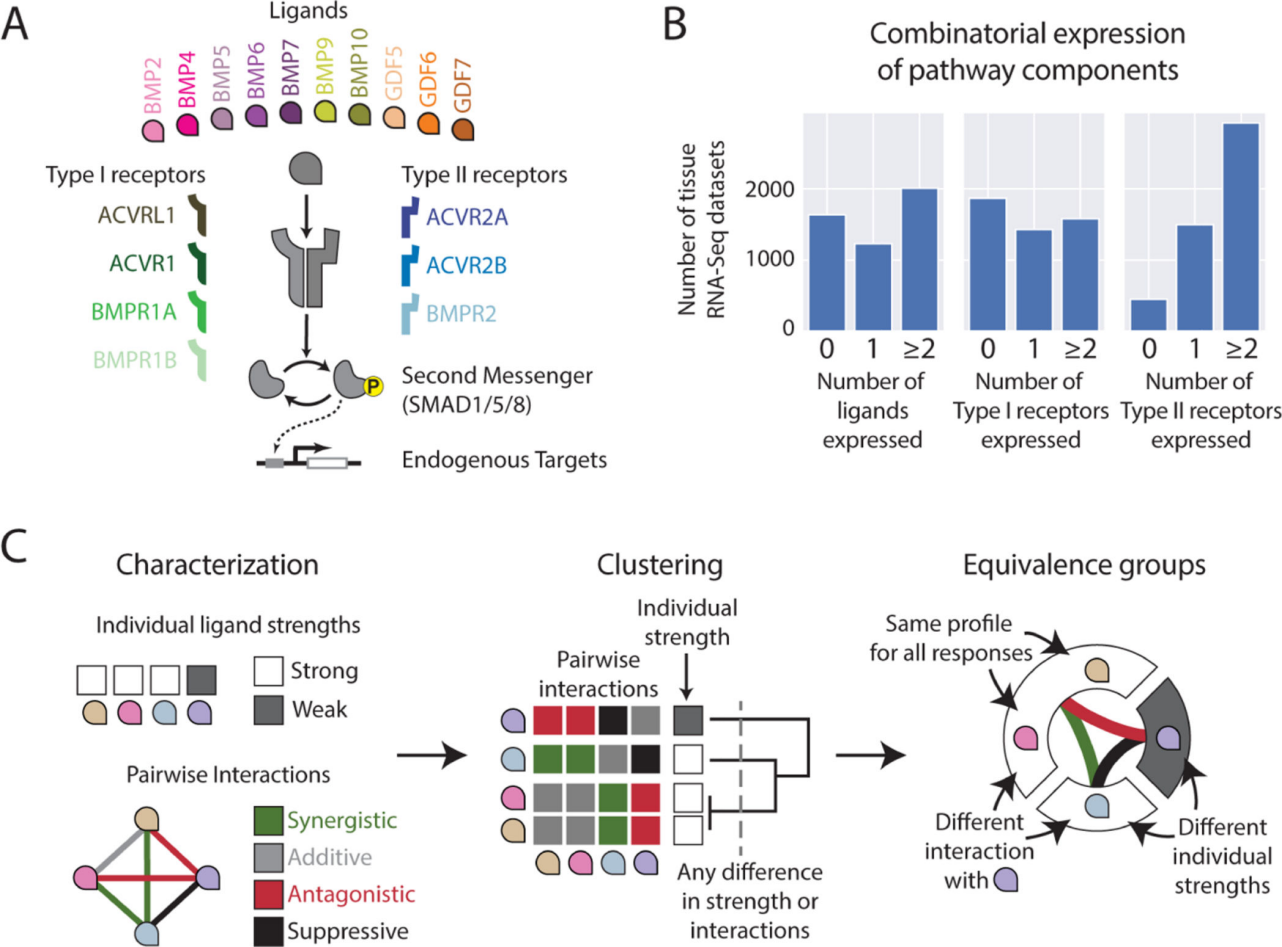
BMP ligands can be classified by their pairwise interactions. A. BMP ligands (colored petal shapes) bind Type I and Type II receptors to form signaling complexes. Active signaling complexes phosphorylate SMAD1/5/8 transcription factors, which translocate to the nucleus to activate endogenous gene targets. Promiscuous ligand-receptor interactions enable the formation of a wide variety of potential signaling complexes. The dimeric nature of the ligands allows each to recruit up to two Type I and two Type II receptors simultaneously. B. Bulk RNA-Seq datasets of various mouse tissues, compiled in (Lachmann et al., 2018), show that BMP ligands, Type I receptors, and Type II receptors are often expressed (i.e. FPKM > 0) as combinations (i.e. 2 or more). C. Systematic measurements of individual and pairwise responses (left) can cluster BMP ligands (middle) into equivalence groups (right). Ligands in the same equivalence group interact in similar ways (synergistic, additive, antagonistic, or suppressive) with other ligands and have the same individual strength.

A prime example of combinatorial and contextual ligand activity can be seen in the Bone Morphogenetic Protein (BMP) pathway. This pathway comprises approximately ten major homodimeric ligand variants, as well as four Type I and three Type II receptors subunits (Figure 1A). Distinct combinations of these components regulate the development of diverse tissues including skeleton, kidney, eye, and brain (Butler and Dodd, 2003; Godin et al., 1999; Huang et al., 2015; Salazar et al., 2016). Interestingly, a given pair of ligands can exhibit either redundant or distinct roles depending on context. For example, BMP9 can replace the essential role of BMP10 in proper vasculature formation, but not in the developing heart (Chen et al., 2013). Similarly, BMP4 can replace BMP7 in the developing kidney, but not in the developing eye (Oxburgh et al., 2005). In addition to variation across tissue context, BMP ligands also have non-overlapping roles when signaling in combinations, as observed in the developing joint (Bandyopadhyay et al., 2006). They can also combine with each other non-additively (Antebi et al., 2017; Klammert et al., 2015; Piscione et al., 1997; Ying et al., 2001). As a result, it remains challenging to understand the roles of different BMP signaling components in a variety of skeletal, respiratory, and brain diseases (Cunha and Pietras, 2011; Salazar et al., 2016; Valer et al., 2019), and to identify therapeutic BMPs for biomedical applications and cell fate control (Carragee et al., 2011; Cicciù et al., 2014; Seeherman et al., 2019).

The primary framework for functionally classifying BMP ligands is based on their receptor preferences. Ligand-receptor affinities govern the formation of signaling complexes, each of which contains a dimeric ligand bound to two Type I and two Type II receptor subunits (Figure 1A). These signaling complexes phosphorylate SMAD1/5/8 effector proteins (as well as non-canonical targets) to regulate downstream genes. While ligands differ in the Type I and Type II receptors that they bind and signal through (David and Massagué, 2018; Shimasaki et al., 2004), receptor preferences as currently understood cannot explain key functional differences between ligands. For instance BMP9 and BMP10 exhibit similar preferences for some receptors (David et al., 2007) but behave non-equivalently, as discussed above (Chen et al., 2013). Moreover, BMP receptors are expressed in combinations (Figure 1B), compete with one another for binding to ligands, and generate complex functional responses to ligand combinations, all of which make it difficult to predict the distribution of signaling complexes, and thereby the overall pathway activity, from qualitative affinity preferences alone (Antebi et al., 2017).

To address these issues, we sought to map the combinatorial effects of ligands, and, more specifically, to identify groups of ligands that function equivalently, across different cell contexts. We define two ligands as equivalent (interchangeable) if they exhibit similar individual activation strengths and interactions with all other ligands. (Analogous to the convention for drug interactions, we will use the term “ligand interactions” to refer to non-additive responses to ligand combinations, without implying direct molecular interaction between the ligands.) To determine ligand equivalence in a given cell context, we can measure responses to BMP pairs and cluster ligands into “equivalence groups” based on the similarity of their pairwise interactions (Figure 1C). Repeating these measurements in different cell types can further reveal how equivalence groups vary between developmental contexts. While previous work identified pairwise responses among a few BMPs, the full set of pairwise interactions is necessary to determine this equivalence structure. (Ligand equivalence groups, defined here, are distinct from equivalence groups in development, which describe groups of cells with the same fate potential (Greenwald and Rubin, 1992).)

This approach was inspired by previous work showing that pairwise analysis of mutations can efficiently reveal the structure of gene modules and protein functions (Costanzo et al., 2016; Schwikowski et al., 2000; Segrè et al., 2005), and that pairwise analysis of antibiotics can similarly classify them into groups with similar biochemical mechanisms of action (Yeh et al., 2006). In these studies, clusters of genes or drugs are defined by patterns in phenotypic, pairwise responses and are shown to emerge from underlying features of the interacting components. Similarly, in the case of BMP signaling, we reasoned that equivalence groups could emerge from subtle or unknown differences in the biochemical properties of different ligands. Equivalence groups could thus empirically distill the functional consequences of unknown parameters, constrain mathematical models of underlying components and interactions, and provide a useful framework for classification and prediction.

Here, we analyzed pairwise combinations of ten of the best-characterized BMP ligands across multiple receptor contexts. This analysis classifies the ten ligands into a smaller number of equivalence groups. Further, we show that these equivalence groups reorganize depending on the receptor expression profile of the signal receiving cell, helping to explain context-dependent effects in development. Finally, to understand how the observed combinatorial ligand responses could emerge from underlying molecular features of the pathway, we fit the full data set to a simplified mathematical model of competitive ligand-receptor interactions. This analysis shows how contextual, combinatorial BMP responses can emerge from the interplay between the affinities that govern signaling complex formation and the specific phosphorylation activities of the resulting complexes. Together, these results reveal the combinatorial and contextual logic of BMP signaling, and provide a framework for understanding other pathways that similarly integrate multi-ligand inputs with promiscuous protein-protein interactions.

## Results

### Quantitative dose-response measurements reveal ligand-specific features

BMPs assemble heterotetramers of Type I and Type II receptors that phosphorylate SMAD1/5/8, which in turn translocates to the nucleus to bind DNA as part of larger transcriptional complexes (Alarcón et al., 2009; Chen et al., 1997; Hata et al., 2000; Ross and Hill, 2008). Here, we quantify the effects of BMP combinations on activation of this canonical SMAD1/5/8-dependent transcriptional response, which plays pivotal roles across a wide range of developmental processes (Hegarty et al., 2013; Lowery and de Caestecker, 2010; Salazar et al., 2016). Non-canonical BMP responses (Blitz and Cho, 2009; Gunnell et al., 2010; Saxena et al., 2018) and the parallel SMAD2/3-dependent TGF-β pathway (Holtzhausen et al., 2014) could also exhibit combinatorial dependence on BMP ligands, but are not investigated here.

To read out SMAD1/5/8 transcriptional activity, we designed a fluorescent reporter containing a fusion Histone 2B (H2B)-mCitrine protein controlled by a synthetic SMAD1/5/8-activated BMP responsive element (BRE) (Korchynskyi and ten Dijke, 2002). The BRE is based on the wild-type Id1 promoter, which responds to BMP, but not TGF-β; depends on the activity of receptors considered specific to BMP (ACVR1, BMPR1A, BMPR1B) but not those specific to TGF-β (ACVR1B, TGFβR1); and exhibits increased responsiveness following the overexpression of BMP-specific SMAD1 and SMAD5 (Korchynskyi and ten Dijke, 2002). BMP ligand addition produced similar fold increases in both YFP expression from the BRE promoter and phosphoSMAD1/5/8 twenty minutes after ligand stimulation (Antebi et al., 2017). Thus, the BRE reporter captures SMAD1/5/8-dependent pathway activation, and allows analysis of cell context-dependent effects that arise downstream of SMAD activation but upstream of gene regulation effects like variation in chromatin state and expression of transcriptional co-factors (Massagué, 2012).

As a base cell line, we selected NAMRU mouse mammary gland (NMuMG) epithelial cells (Figure S1A), which can respond to BMP signals without differentiating (Piek et al., 1999), and express five of the seven BMP receptors: ACVR1, ACVR2A, ACVR2B, BMPR1A, and BMPR2 (Antebi et al., 2017). NMuMG cells stably expressing the BRE reporter exhibited unimodal distributions of YFP fluorescence levels that responded in a dose-dependent manner to varying concentrations of BMP ligands (Figure S1B), allowing quantitative readout of pathway activity in response to ligands (Figure S1C).

We identified ten homodimeric BMP ligands that play pivotal roles in development and are known to activate SMAD1/5/8. (Hinck et al., 2016; Weiss and Attisano, 2013) (Figure 1A). This core set omits BMP heterodimers, which have been shown to produce higher levels of pathway activation per unit mass than homodimers (Aono et al., 1995; Israel et al., 1996; Miao et al., 2018; Morimoto et al., 2015; Sun et al., 2012; Suzuki et al., 1997; Valera et al., 2010) and whose physiological relevance is increasingly recognized (Butler and Dodd, 2003; Dutko et al., 2021; Kim et al., 2019; Tillet et al., 2018). These 10 homodimers exhibited diverse dose-response characteristics (Figure S1D). Seven strongly activated the pathway with various saturating levels, concentrations for half-maximal activation (EC50), and logarithmic sensitivities, quantified by a fitted Hill coefficient. EC50 values ranged over three orders of magnitude (Figure S1E), while the Hill coefficient, quantifying the ultrasensitivity of the dose-response, varied between values of 1 and 2 (Figure S1F). We defined the Relative Ligand Strength (RLS) as the pathway activity at saturating ligand concentrations normalized by the activity of the strongest ligand, and quantified it for all 10 ligands. RLS values fell into three distinct tiers (Figure S1G). Most BMPs activated the reporter at high and comparable levels. However, others produced only half as much activation (BMP10) or barely activated the reporter above background (GDF5, GDF6, GDF7), even at high doses. As shown below, low responsiveness was not indicative of inactive protein, as the GDFs did activate cells with ectopic expression of the GDF-responsive receptor BMPR1B (Figure S6E) (Nishitoh et al., 1996).

### Ligands exhibit diverse combinatorial profiles

**Figure 2:**
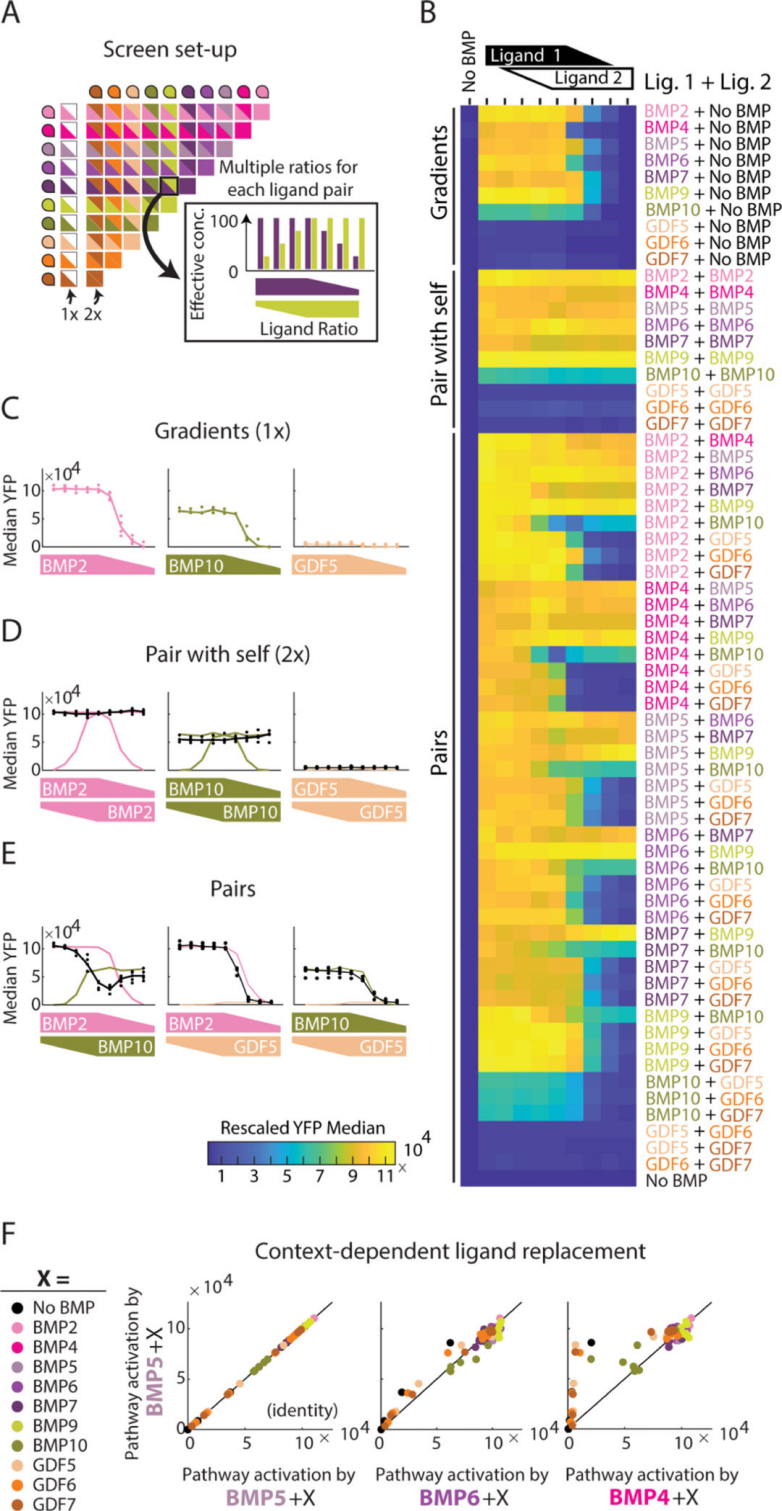
Pairwise titration of relative ligand concentrations reveals ligand interactions. A. Fully classifying ligand equivalence groups requires analysis of all possible pairs, as well as measurements of individual activity (1X) and each ligand paired with itself (2X) as a positive control. Responses in each category are sampled at multiple ratios (inset), with at least one ligand very near its saturating concentration in each ratio. B. The full dataset includes measurements for all possible gradients, pairs, and pairs with self, which are shown as each row of the heatmap. The columns correspond to concentration ratios, with the leftmost column reserved for a no BMP control. Medians of four biological repeats are shown with heatmap colors. C. Sample gradients of BMP2, BMP10, and GDF5 show the diversity of individual ligand strengths. Dots are four biological replicates, and the line connects the median for each concentration. The ligand concentrations are high and saturating on the left (five points total) and a titration to no BMP on the right, as shown by the trapezoid height on the x-axis. D. Ligands paired with themselves show saturated additive relationships. The colored lines shows individual ligand responses as in (C), whereas the black line and dots show the response to the pair indicated on the x-axis, either BMP2, BMP10, or GDF5 paired with itself for multiple concentration ratios. E. All possible pairs of three ligands (BMP2, BMP10, and GDF5) show various pairwise interactions. Responses to pairs can be lower than both individual ligands (left) or interpolate between the two (middle and right), though GDF5 antagonizes BMP10 (right) to a much lesser extent than it antagonizes BMP2 (middle). The colored lines show median individual ligand responses as in (C), whereas the black dots and line are the response to the pair. The concentration ratios are shown using the same trapezoids on the x-axis. F. Responses to BMP5 paired with another ligand (“X”, colors) are plotted against the response to other ligands (BMP5, BMP6, or BMP7) also paired with X. Since all pairs are sampled at seven ratios, there are seven dots for each X (i.e. color). The first two plots show that BMP6 is a nearly equivalent replacement for BMP5, as BMP5+X closely resembles BMP6+X (i.e. dots are close to the y=x line). By contrast, BMP4 produces much lower activation than an equivalent amount of BMP5, in the presence of BMP10, GDF5, GDF6, or GDF7. Pathway activation is the median of four biological repeats, quantified by flow cytometry.

Two ligands of similar strength may differ in their combinatorial interactions with other ligands. Based on previous work showing that pairwise interactions between BMPs vary with ligand ratio and generally increase with concentration (Antebi et al., 2017), we reasoned that scanning ligand ratios at high concentrations of at least one ligand could efficiently identify interactions with a smaller number of measurements compared to analysis of a full double ligand titration. (We note, however, that this approach could miss interactions appearing only at lower BMP concentrations.) Therefore, each ligand pair was observed at nine distinct ligand concentration ratios, with absolute concentrations spanning the ligand’s input dynamic range (Figure 2A, inset). Altogether, we measured the responses to all 45 pairs of the 10 ligands (Figure 2A), each ligand alone, and, as a reference, each ligand paired with itself, for a total of 660 measurements per experiment. We programmed a robotic liquid-handling system to enable semi-automated measurements of all these ligand combinations in a single experiment (STAR Methods), with responses quantified by flow cytometry after 24h incubation with the ligands (Figure 2B), exhibiting an array of responses for individual ligands, self-pairs, and true pairs (e.g. Figures 2C-E).

Analysis of the resulting data revealed that two ligands with similar individual activity can behave differently in the presence of another ligand. For example, BMP4, BMP5,and BMP6, all activated strongly (RLS~1). Replacing BMP5 with BMP6 produced no change in pathway activity, including in the presence of each other ligand, and at all concentration ratios, indicating that these two ligands behave nearly as equivalently as observed when trivially combining BMP5 with itself (Figure 2F, left and middle). By contrast, replacing BMP5 with an equivalent dose of BMP4 altered signaling activity in the presence of BMP10 or any of the GDFs (Figure 2F, right). Thus, ligands that exhibit similar individual activity (RLS) are not necessarily equivalent when considered in combination with other ligands.

### Ligand interactions reveal BMP equivalence groups in NMuMG cells

Systematically mapping ligand equivalence requires a quantitative scale to measure ligand interactions. With drug interactions and gene epistasis, one typically quantifies deviations from an “additive” or non-interacting expectation (null hypothesis) given by the product of the effects of the individual perturbations (Bliss, 1939; Loewe, 1928; Russ and Kishony, 2018; Yeh et al., 2006). These frameworks do not apply in a straightforward way to signaling, where the additive expectation for a pair of ligands depends on their concentrations relative to the point of saturation. To see why, consider two perfectly identical ligands. At sub-saturating concentrations in a linear region of the dose-response curve, their combination is expected to generate a response equivalent to that produced by twice the concentration of either ligand. However, in the opposite extreme, where both are at saturation, the same two identical ligands would combine to produce an activity no greater than that generated by either ligand individually. Further, because two ligands can produce different maximal activation levels, they can generate similar activity even if one is saturating and the other is not. As a result, the additive expectation for two ligands can, in general, range from the maximum to the sum of their individual activities.

**Figure 3:**
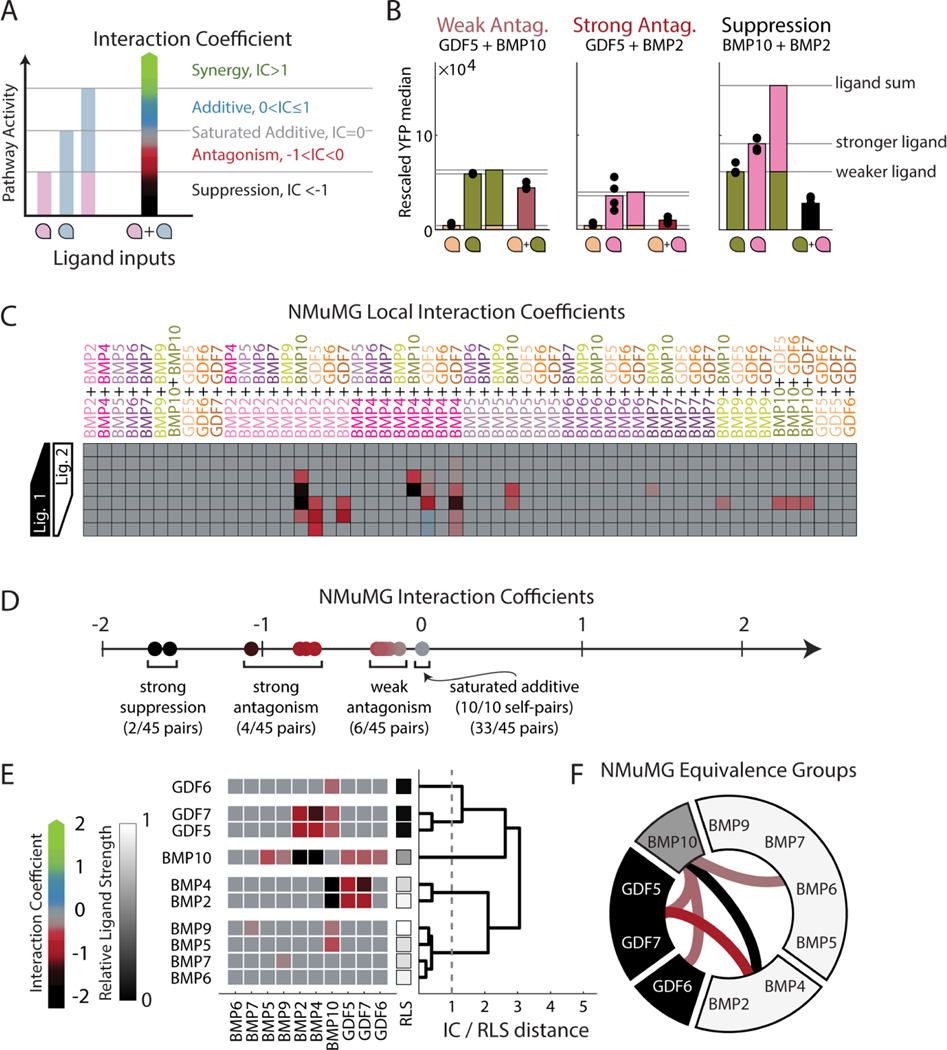
Antagonism and suppression define BMP equivalence groups in NMuMG cells. A. The Interaction Coefficient (IC) classifies different regimes of pairwise responses by comparison to three references (horizontal lines): the two individual ligand responses, *f(A)* and *f(B)*, and the sum of those responses, *f(A) + f(B)*. The behavior type and strength for a given pairwise response, *(A+B)*, is indicated by its IC value and associated color map: black for suppressive, red for antagonistic, gray for saturated additive, blue for additive, and green for synergistic. IC values linearly interpolate between reference points. Though IC has no upper bound, values rarely exceed 2, which is the upper limit shown on all plots. See Figure S2A and STAR Methods for IC formula. B. Pairwise interactions of different strengths (e.g. weak versus strong antagonism) as well as different types (e.g. antagonism versus suppression) occur in NMuMG. Each subplot highlights a different ligand pair at the ligand concentrations and ratio that produced the largest non-zero Interaction Coefficient. Within each subplot, dots are biological replicates, and bars are the median, showing (left to right) weaker and stronger individual responses (colored by the ligand) followed by the pairwise response (colored by IC colormap as in Figure 3A). The horizontal lines mark the three references that IC is calculated relative to: the weaker and stronger individual responses followed by their sum. C. Interaction Coefficients were calculated at each ligand ratio for each ligand pair and are plotted with the same colormap as in (A). The classification for each pair is the largest nonzero IC value across all concentration ratios. D. Across 10 self-pairs and 45 pairs, the majority of responses are saturated additive. The remainder are weakly antagonistic, strong antagonistic, or suppressive, with no additivity or synergy. E. Hierarchical clustering of IC and RLS values in NMuMG cells reveals five distinct equivalence groups, defined to separate ligands with similar pairwise interactions and individual strengths (STAR Methods). The square colors indicate either the ligand’s IC value (see colormap in (A)) when paired with another ligand or the RLS grayscale for its individual strength (cf. Figure S1G), as indicated by x-tick labels. The dendrogram on the right-hand side shows complete-linkage clustering of Euclidean distance between ligand features. The RLS and IC values are weighted such that a distance of 1, the cut-off to define clusters, indicates a significant difference in either individual strength or a qualitative difference in one pairwise interaction between two ligands (STAR Methods). F. BMP ligand equivalence in NMuMG cells, as classified by hierarchical clustering in (E), comprises five equivalence groups. Wedge grayscale value shows approximate RLS value of the contained ligands, while connecting line colors represent pairwise interactions between ligand groups. Groups without a linking edge have a saturated additive interaction. Ligands with similar individual strengths can be grouped differently based on their pairwise interactions. See also Figures S1, S2.

An ideal ligand interaction metric would distinguish different pairwise response types solely by comparing them to a null hypothesis represented by the interaction of the ligand with itself, thereby avoiding additional assumptions about the nature of additivity. To this end, we defined the Interaction Coefficient (IC) (Figures 3A, S2A, STAR Methods), which maps different interaction types onto a single linear scale by comparing the activities of the two ligands individually and in combination. The IC scale captures the full range of additive behaviors that occur when a ligand is combined with itself within the interval from IC=0 (fully saturated additivity) to IC=1 (linear additivity). IC values outside this interval represent non-additive interactions. Specifically, negative IC values indicate antagonistic interactions, in which the combined activity is weaker than the saturated additive response. IC values below −1 represent the suppressive case where the combined activity is weaker than that of both individual ligands. On the positive side, IC values greater than 1 indicate positive synergy, where the combined activity of the two ligands exceeds the unsaturated additive response. The resulting scale can be written as a set of piecewise-linear equations (Figure S2A). To classify interactions reliably despite experimental noise, we required that deviations from additivity exceed the variation between technical replicates (Figure S2B, STAR Methods). Note that, despite its different definition, the IC categories — synergistic, antagonistic, and suppressive—mirror those used to study drug interactions (Bollenbach et al., 2009; Yeh et al., 2006). They also can be related to a previous classification of two-ligand response types, including balance, ratiometric, and imbalance functions, respectively (Antebi et al., 2017).

We next applied the IC scale to analyze the distribution of interactions in NMuMG cells (Figures 3B,C, S2C), with manual corrections in a few cases of unusually large or small variance (Figures S2D-F). As expected, all “self pairs” measured at saturating concentrations exhibited an IC value of 0 corresponding to saturated additivity (Figure 3D); deviations from these values (i.e., nonzero IC values) thus indicated a deviation from the null hypothesis of how a ligand interacts with itself. Among pairs of distinct ligands, a majority were saturated additive (Figure 3C, gray squares), with others showing antagonism (red) or suppression (black) (Figures 3D, S2C).

We next combined the full set of pairwise interactions with measurements of individual ligand strength and clustered ligands into equivalence groups (Figure 3E,F). Ligands appearing in the same equivalence group (cluster) exhibited the same pattern of individual strengths and interactions with other ligands, suggesting they function interchangeably across pairwise ligand combinations (Figure 3E). To reduce sensitivity to ligand differences resulting from measurement noise or subsampling of concentrations, we imposed an additional requirement for non-equivalence. Specifically, we required a difference of at least 1 in IC values, corresponding to a qualitative change in interaction type, or a difference of at least 0.5 in RLS, indicating a large difference in individual strength regardless of any pairwise differences. To efficiently visualize the equivalence groups and their relationships, we represented them around a circle (Figure 3F), where the grayscale level of each group indicates the mean activity of its constituent ligands and internal lines represent the non-additive interactions between groups, colored by interaction type.

This representation shows that the 10 ligands comprise five distinct equivalence groups in NMuMG cells: [BMP2, BMP4], [BMP5, BMP6, BMP7, BMP9], [BMP10], [GDF5, GDF7], and [GDF6], with five non-additive interactions between those groups. (Here and below, square brackets denote sets of ligands that constitute an equivalence group.) The group [BMP5, BMP6, BMP7, BMP9] activated strongly as individuals and interacted additively with one another and all other ligands, except for weak antagonism with BMP10. The second group, [BMP2, BMP4], also contained strong activators, but was distinguished by its susceptibility to antagonism by GDF5 and GDF7 and suppression by BMP10. The third and fourth groups, [GDF5, GDF7] and [GDF6], comprised non-activating ligands that respectively did or did not antagonize [BMP2, BMP4]. Finally, [BMP10] formed its own equivalence group based on both its unique pattern of interactions and its intermediate individual strength. Thus, many of the ligand distinctions represented here emerge only from pairwise interactions and could not have been inferred from analysis of individual ligands.

### Ligand equivalence depends on cell context

The equivalence map determined for NMuMG cells need not be universal to all cell types. Changes in receptor expression can alter the type of pairwise ligand interaction (Antebi et al., 2017), and BMP receptor expression is known to vary among cell types in developing tissues (Danesh et al., 2009). Therefore, we next asked how equivalence groups vary with cell context in general and with receptor expression profiles more specifically.

**Figure 4:**
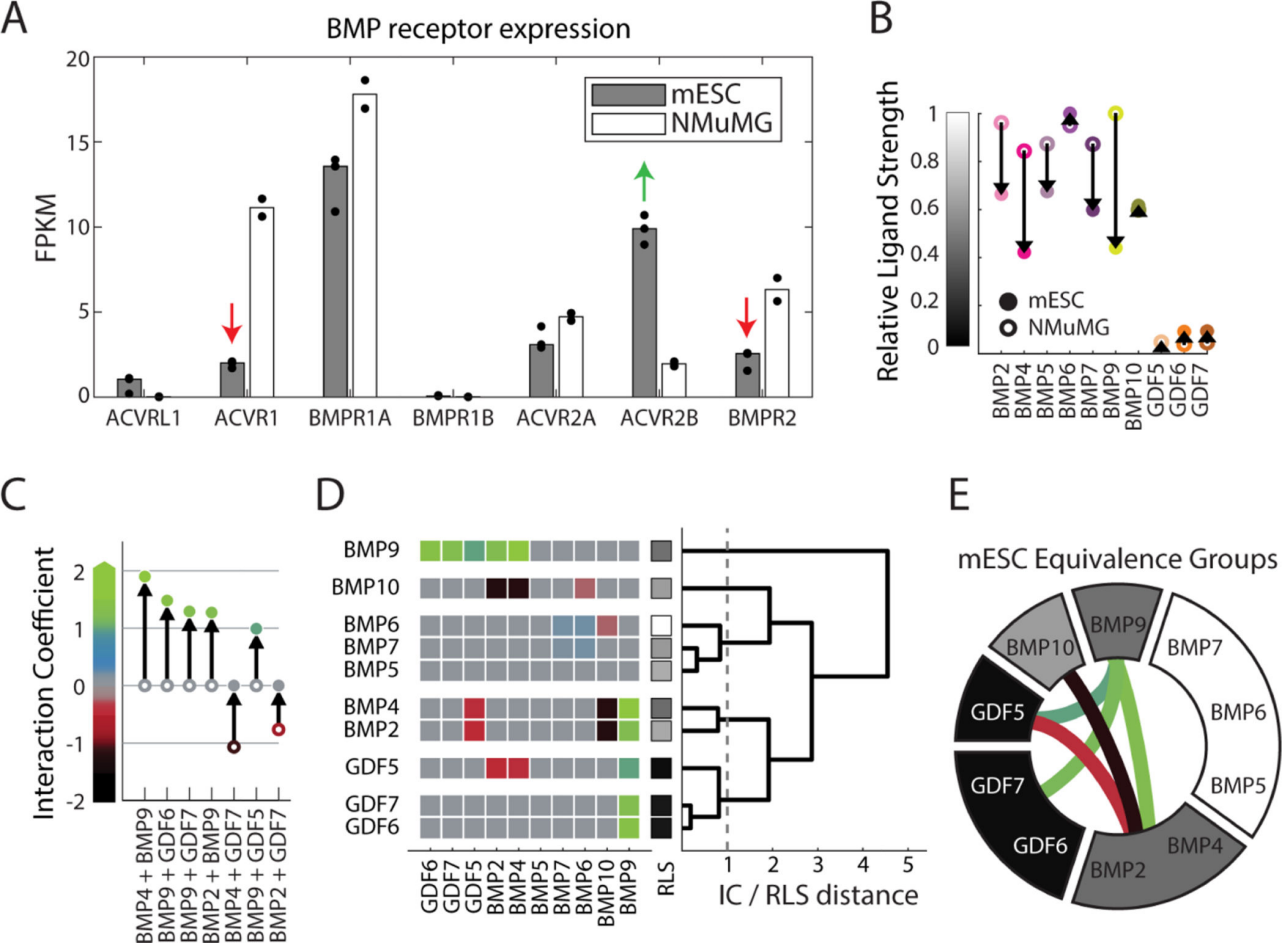
Mouse ES cells exhibit distinct BMP equivalence groups. A. RNA-Seq of BMP receptors in mESC reporter and wild-type NMuMG cells show differences in receptor expression (highlighted by green and red arrows) between the two cell lines, with mESCs expressing more ACVR2B, but less ACVR1 and BMPR2. Dots show at least two biological repeats, and bar height is median. B. RLS values in mESCs (closed circle) differ from NMuMG cells (open circle, cf. Figure S1G), with more intermediate strength activators in mESCs. Arrows indicate the change in mESCs relative to NMuMG cells. C. IC values for seven pairwise interactions changed a large amount (|ΔIC|>0.5) between NMuMG cells (open circle) and mESCs (closed circle). The majority show a net increase of IC, producing five new synergistic interactions. Both the color and y-axis value of the open and closed circles indicate the IC value for the indicated cell lines, computed on the median of four biological repeats. D. Hierarchical clustering of IC and RLS values in mESCs reveal a distinct set of ligand equivalence groups compared to NMuMG cells (cf. Figure 3E), using the same cutoff criteria to group ligands by similar individual strengths and pairwise interactions (STAR Methods). The square colors indicate either the ligand’s IC value when paired with another ligand or the RLS grayscale for its individual strength (see y-axis colormaps in (B) and (C)), as indicated by x-tick labels. The dendrogram on the right-hand side shows complete-linkage clustering of Euclidean distance between ligand features. E. BMP equivalence in the mESC reporter, as classified by hierarchical clustering in (D), includes six equivalence groups. Grayscale values indicate the approximate RLS of the grouped ligands, while connecting line colors indicate the pairwise interactions between groups of ligands. Groups without a linking edge have a saturated additive interaction. See also Figure S3.

Mouse embryonic stem cells (mESCs) present an ideal cell context in which to study BMP signaling. Manipulation of BMP signaling in mESCs is a key step in many directed differentiation protocols (Craft et al., 2013; Gouon-Evans et al., 2006), and single BMPs and pairs can produce distinct cell fates in the same protocol (Andrews et al., 2017). Here, we used a fluorescent mESC reporter line, based on E14 mESCs, to analyze the dose response for each of the ten ligands tested previously in NMuMG cells (STAR Methods). The short timescale of our experiments (<24h) and lack of additional (i.e. non-BMP) components required for most differentiation protocols allows us to make quantitative dose response measurements without a large effect on cell state or fate. The E14 mESCs used in this study also differ from NMuMG cells in their expression of three of the five BMP receptors, providing a different BMP receptor context (Figure 4A). Finally, NMuMG and E14 cells are known to respond in distinct ways to some ligand combinations (Antebi et al., 2017).

The dose response measurements reveal that the mESC reporter responded differently to the individual ligands compared to NMuMG cells. They exhibited a greater diversity of activation strengths (Figure 4B), as well as greater EC_50_ values and lower Hill coefficients, indicating larger input dynamic ranges with lower sensitivity to moderate ligand concentrations (Figure S3A). In fact, high concentrations (approaching 10 μg/mL) of some ligands failed to fully saturate the response.

Among the responses to pairs of BMPs, a subset showed marked differences (|ΔIC|>0.5) between the two cell lines (Figure 4C), though most pairwise interactions remained saturated additive (Figures S3B-D). Most notably, BMP9 acquired synergistic interactions with activating ligands [BMP2, BMP4] as well as with non-activating ligands [GDF5, GDF6, GDF7] (Figure S3E). This synergy was not a side-effect of the slightly subsaturating ligand concentrations used in mESCs. While certain combinations of BMP2 with BMP9 could produce pairwise responses clearly exceeding the sum of their individual responses in mESC (Figure S3F-H), no combination of BMP2 with BMP9, even at sub-saturating concentrations, produced a similarly synergistic response in NMuMG cells (Figure S3I-K). Rather, all pairwise responses to BMP2 and BMP9 overlapped with the stronger individual ligand response in NMuMG (Figure S3K). Such synergistic interactions were previously proposed to emerge through a mechanism in which competition between ligands for receptor binding redistributes ligands to higher activity complexes compared to those that would form in the presence of a single ligand (Antebi et al., 2017). In parallel with the new synergies, strong antagonistic interactions between the GDF ligands and [BMP2, BMP4] were lost in the mESCs.

Thus, we observed an overall shift from antagonism to synergy across ligand pairs (Figure 4C). Combined with the changes in individual ligand strength, this shift generated distinct ligand equivalence relationships (Figure 4D). While some groups and interactions were preserved, such as the suppression between [BMP2, BMP4] and [BMP10], many other groups were split or merged (cf. Figures 4E, 3F). Together with the map of equivalence in NMuMG, these results provide two key findings. First, most ligand pairs interact in a (saturated) additive manner, but do not necessarily function equivalently in a single cell line when one considers both their individual strength and their interactions with other ligands. Second, ligand equivalence groups are, in general, contextual (i.e., differing among cell types), such that two ligands may function equivalently in one cell type but not in another.

### ACVR1 knockdown recapitulates features of mESC ligand interactions

We next sought to better understand how ligand equivalence can vary between cell contexts. Differences in responsiveness to BMPs can in principle emerge at multiple points between ligand presentation and SMAD1/5/8 activation (Massagué, 2012; Massagué and Chen, 2000). For example, the number of ligand-receptor signaling complexes that form in the presence of a specific ligand input depends on how many and which type of BMP receptors are expressed on the cell membrane, as well as the presence of extracellular inhibitors and co-receptors (Babitt et al., 2005; Balemans and Van Hul, 2002; Cheifetz et al., 1992; Yilmaz et al., 2016). The activity of these signaling complexes can further depend on inhibitory SMADs and competition for shared SMAD4 (Lucarelli et al., 2018; Miyazawa and Miyazono, 2017). Moreover, other aspects of culturing conditions, such as tight packing of cells in mESC colonies, may alter subcellular localization of receptors and affect BMP responsiveness. Nevertheless, because receptors directly interact with ligands, exert the earliest influence on ligand integration, and are responsible for differential BMP responsiveness in diverse processes, they represent the strong candidate for controlling combinatorial ligand responsiveness (Crisan et al., 2015; Danesh et al., 2009; Panchision et al., 2001; Yamauchi et al., 2008). Without excluding effects at other levels, we therefore focused here on the role of receptor expression in combinatorial ligand perception.

The E14 mESCs used in this study express less ACVR1 and BMPR2, but more ACVR2B, than NMuMG cells (Figure 4A). Out of these three receptors, we first focused on ACVR1, a Type I receptor that is expressed in a variety of cell types and plays key roles in bone and brain diseases (Agarwal et al., 2017; Shore et al., 2006; Valer et al., 2019), and asked whether reducing its expression could make ligand responses in NMuMG cells more similar to those of mESCs. To achieve stable knockdown of ACVR1, we constitutively expressed shRNA against ACVR1 in the NMuMG reporter cell line. This perturbation reduced expression to less than 20% of its unperturbed expression level with minimal effects on off-target receptors (Figure S4A). Its effects on ligand interactions were similar to those observed independently in response to transient siRNA knockdown of ACVR1 (Figure S4B).

**Figure 5:**
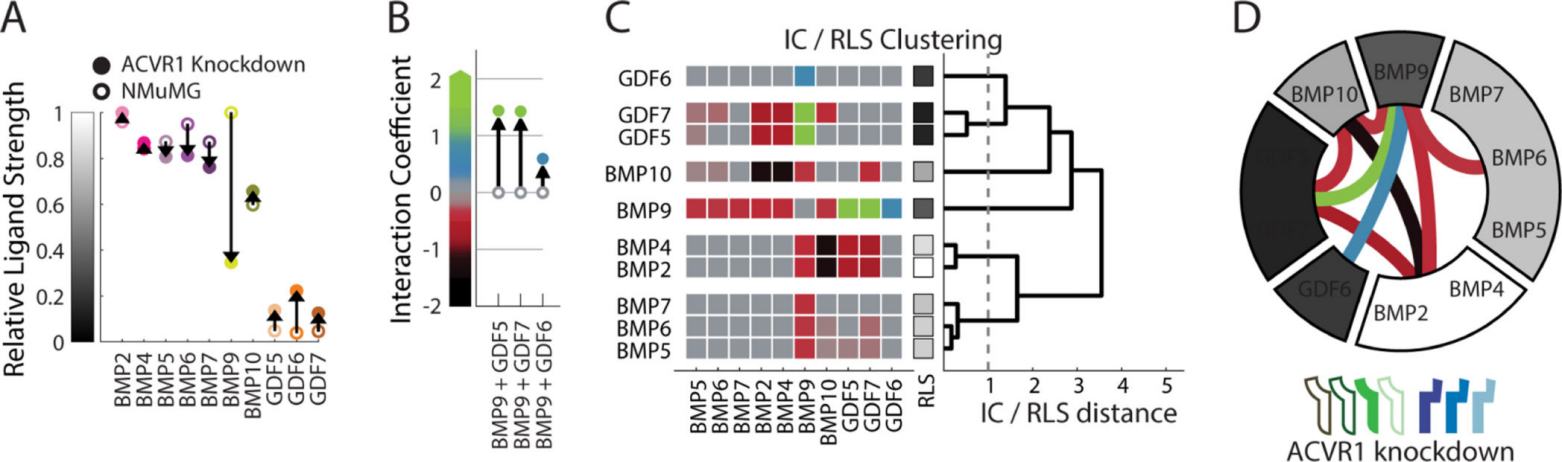
ACVR1 knockdown reveals the effects of receptor context on ligand equivalence. A. ACVR1 knockdown (closed circle) alters RLS values compared to NMuMG cells (open circle, cf. Figure S1G). BMP9 is the most strongly affected. B. Three pairwise interactions changed a large amount (|ΔIC|>0.5) between NMuMG cells (open circle) and ACVR1 knockdown (closed circle), producing new synergistic interactions with BMP9. Open and closed circles are colored by IC value. C. Hierarchical clustering of RLS and IC values (as in (A) and (B)) in the ACVR1 knockdown context shows a more complex equivalence map. The square colors indicate either the ligand’s IC value when paired with another ligand or the RLS value for its individual strength (see y-axis colormaps in (A) and (B)), as indicated by x-tick labels. The dendrogram on the right-hand side shows complete-linkage clustering of Euclidean distance between ligand features. D. BMP equivalence following ACVR1 knockdown, as classified by hierarchical clustering in (C), includes six groups. Wedge colors show approximate RLS value of the contained ligands, while connecting line colors indicate pairwise interactions between groups of ligands. Groups without a linking edge have a saturated additive interaction. The receptor perturbation is shown as a cartoon of filled and empty receptors, which are drawn in the following order: ACVRL1, ACVR1, BMPR1A, BMPR1B, ACV2A, ACVR2B, BMPR2. See also Figure S4.

ACVR1 knockdown recapitulated some of the observed differences between NMuMG cells and mESCs. BMP9, the strongest individual activator of NMuMG cells and a ligand known to signal through ACVR1 (Valer et al., 2019), signaled only weakly in the knockdown cells, consistent with its low activity in mESCs (Figure 5A, cf. 4B). ACVR1 knockdown also reproduced the synergy of BMP9 with GDF5, GDF6, and GDF7 observed in mESCs (Figures 5B, S4G,H, cf. 4C). Finally, in both mESCs and ACVR1 knockdown, the equivalence of BMP9 with BMP5, BMP6, and BMP7 was eliminated (Figures 5C,D, cf. 4E). By contrast, other distinct features of the mESC response did not appear in the ACVR1 knockdown. For example, [BMP2, BMP4] were still antagonized by GDF7, and [BMP2, BMP4] were not synergistic with [BMP9] (Figure 5C). ACVR1 knockdown also produced new interactions not observed in NMuMG cells or mESCs. [BMP9] antagonized, rather than synergized with, the other strongly activating ligands, including [BMP2, BMP4] and [BMP5, BMP6, BMP7]. This result shows that a single ligand (BMP9, in this case) can produce interactions of opposite signs in the same cell context. Lastly, while the majority of the pairwise interactions conserved across all three cell lines were saturated additive, they also included GDF5’s antagonism of [BMP2, BMP4] and [BMP10]’s suppression of [BMP2, BMP4] (Figure S4F, cf. Figures 5D, 4E, 3F). More generally, these results suggest that differences in receptor expression, and ACVR1 expression specifically, are sufficient to generate dramatic changes in combinatorial ligand responses, and could account for some of the distinct ligand responses of mESCs and NMuMG cells.

### Perturbations of multiple BMP receptors reveal flexibility of ligand equivalence

To gain further insight into how individual receptors could modulate ligand equivalence, we perturbed four additional BMP receptors, two that broadly interact with many ligands, and two that exhibit stronger ligand preferences. Specifically, we knocked down each of the two most abundant receptors in NMuMG cells, BMPR1A and BMPR2, and ectopically expressed each of the two receptors with the weakest endogenous expression in NMuMG cells, ACVRL1 and BMPR1B. These four receptors differ widely in their ligand selectivity and expression profiles. BMPR2 and BMPR1A bind a diverse set of BMPs (Nishitoh et al., 1996; Orriols et al., 2017; Ten Dijke et al., 1994) while ACVRL1 and BMPR1B have more specific ligand preferences (David et al., 2007; Nishitoh et al., 1996). Additionally, BMPR1A and BMPR2 are broadly expressed across distinct tissues, whereas BMPR1B and ACVRL1 are highly expressed in relatively few tissues (Cunha and Pietras, 2011; Danesh et al., 2009).

**Figure 6:**
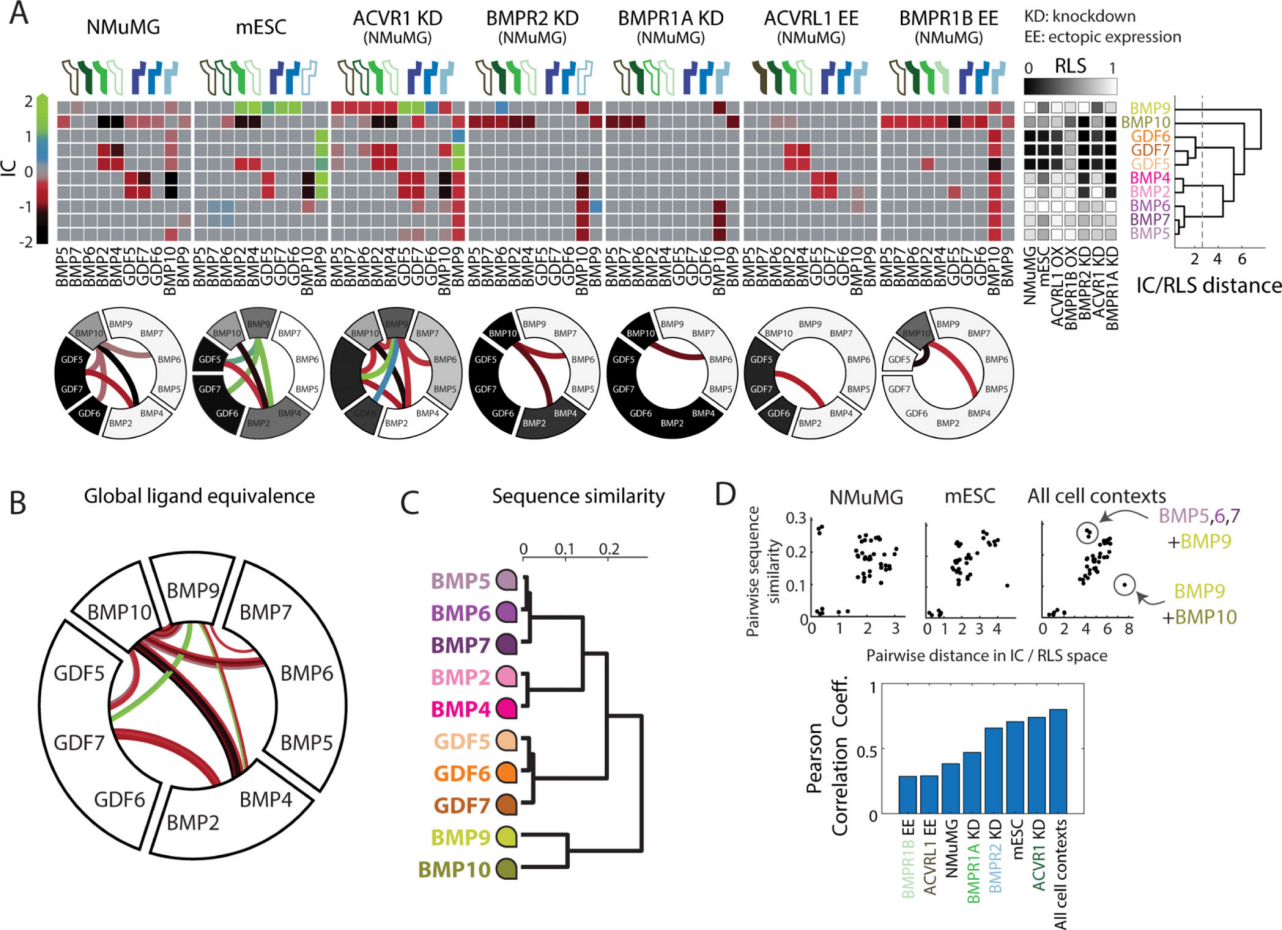
Analysis of multiple cell contexts reveals global ligand equivalence groups. A. Clustering RLS and IC values for all ligands individually and in pairs across all cell contexts shows global ligand similarity (upper row). The full set of IC values show the distribution of ligand interaction types (colors as in Figure 3E) and identify ligands with different frequencies of non-additive interactions. The lower row shows the corresponding ligand equivalence groups (first three reproduced from Figures 3F, 4E, 5D). For the dendrogram (right), clustering was done by complete linkage of the Euclidean distance between RLS and IC features, as before. B. The global equivalence map was determined by cutting the dendrogram (right side of (A)) at the indicated distance (STAR Methods). Internal links show all interactions between those ligands that are not saturated additive. Global groups tend to have linkages of the same interaction type (i.e. similar colors), though with widely varying frequencies. For example, BMP2 and BMP4 almost always interact with BMP10 but never with BMP5. C. Recombinant ligand sequences were clustered by complete-linkage clustering of pairwise alignment distance, as computed by the BLOSUM50 matrix between globally-aligned sequences, neglecting all gaps. Ligands comprise roughly four groups of related proteins, indicated by color families. The recombinant ligands used in this study constitute the mature secreted form of the ligand, exclude pro-domains, and vary in length from 108 to 146 amino acids (see also Figure S7A). D. Sequence similarity, quantified as in (C), correlates with IC/RLS similarity observed in each cell context, though the extent of correlation varies. In the correlation across all cell contexts, notable outliers include BMP9’s surprising functional difference from BMP10 despite their significant sequence similarity, and BMP9’s unexpected similarity with BMP5, BMP6, BMP7, despite relatively dissimilar sequences. Pearson correlation coefficient (bottom) quantifies correlation between sequence similarity and IC/RLS distance for each cell line. Highest correlation is achieved by considering all cell contexts together. See also Figure S5, S6, and S7.

For knockdown of BMPR1A and BMPR2, we generated stable shRNA knockdown cell lines similar to the ACVR1 line (Figures S5A,B). Knocking down BMPR2, which is also reduced in mESCs relative to NMuMG cells, decreased activation by BMP2 and BMP4 and removed their antagonism by GDF5 and GDF7, reducing the number of equivalence groups to four (Figures 6A “BMPR2 KD,” S5C-G). Thus, BMPR2 knockdown recapitulated distinct aspects of the equivalence relationships observed in mESCs compared to those produced by ACVR1 knockdown. Knockdown of BMPR1A also simplified the equivalence structure, reducing activation by BMP2 and BMP4 and including only one non-additive interaction among two of the three groups (Figures 6A “BMPR1A KD,” S5H-J). The loss of most non-additive interactions in BMPR2 and BMPR1A knockdown cells suggests that these receptors, unlike ACVR1, are necessary for many non-additive ligand interactions in NMuMG cells. More generally, it shows that the BMP equivalence structure can be simpler than that observed in wild-type NMuMG and mESC cell lines.

In contrast to the relatively promiscuous receptors perturbed so far, the receptors ACVRL1 and BMPR1B are known to be more ligand-specific (Chai et al., 2020; Cunha and Pietras, 2011; Nishitoh et al., 1996), primarily binding BMP9 and BMP10 or the GDFs, respectively. To explore their effects on ligand interactions, we stably and ectopically expressed them by integrating constructs encoding receptor cDNA into the NMuMG reporter cell line, where they are not normally expressed (Figure S6A). Both perturbations reduced the number of equivalence groups and non-additive interactions. Ectopic ACVRL1 expression increased activation by BMP10 and removed its many suppressive and antagonistic interactions with other ligands (Figure 6A “ACVRL1 EE,” S6B-D). Similarly, BMPR1B increased activation by its preferred ligands GDF5, GDF6, GDF7, and removed their antagonism of BMP2 and BMP4 (Figure 6A “BMPR1B EE,” S6EH), allowing 8 of the 10 ligands to signal equivalently. Thus, ectopic expression of each receptor resulted in its preferred ligands becoming equivalent to other strongly activating, weakly interacting ligands, such as BMP5, BMP6, BMP7, and BMP9.

### Comparing signaling across cell contexts reveals global structure of ligand equivalence

Together, the set of pairwise interactions and corresponding equivalence maps observed across all cell contexts studied in this work provide a more global view of the structure of BMP signaling than those obtained from any single context alone and reveal the plasticity of both ligand strength and interactions (Figure 6A, top). While most pairwise interactions were additive, every ligand participated in synergy or antagonism with another ligand in at least one receptor context. Further, many ligands, including BMP2 and BMP4, were active only in some but not all receptor contexts. Despite the overall plasticity, certain trends were broadly conserved across contexts. GDF5, GDF6, and GDF7 were usually weak activators, while BMP5, BMP6, and BMP7 strongly activated all cell types. Non-additive interactions tended to be consistent for a given ligand pair, exhibiting the same interaction type (i.e., color) across cell lines. The only exceptions were the possibility of both synergistic and antagonistic interactions between [BMP2, BMP4] and [BMP9], as well as both antagonistic and suppressive interactions between [BMP2,BMP4] and [BMP10]. Finally, certain ligands dominated certain interaction types. All synergistic ligand interactions involved BMP9, whereas all suppressive interactions involved BMP10. Future analysis of additional contexts could reveal additional plasticity beyond that analyzed here. Thus, this global analysis shows that, considered as a system, the meaning of any given BMP ligand is inherently contextual, depending both on other ligands and on receptor context.

To represent the full dataset in a more compact way, we clustered ligands according to the similarity of their individual strengths and interactions across all receptor contexts (Figure 6A, right). This approach classified the ten BMPs into five *global* equivalence groups: [BMP2, BMP4], [BMP5, BMP6, BMP7], [BMP9], [BMP10], and [GDF5, GDF6, GDF7] (Figure 6B, STAR Methods). [BMP2, BMP4], as well as [BMP5, BMP6, BMP7], exhibited the strongest version of equivalence. Despite frequent changes in individual or combinatorial signaling across cell contexts, responses within each of these groups always changed in concert, such that the ligands in each group remained equivalent across all contexts. This global equivalence map also depicts the prevalence and variability of ligand interactions (Figure 6B). For example, the mixture of red and green lines between [BMP9] and [BMP2, BMP4] indicates that their interactions can vary from synergy to antagonism. In all receptor contexts except one, [BMP10] interacts non-additively with [BMP2, BMP4], while [BMP5, BMP6, BMP7] exhibits only additive interactions with [BMP2, BMP4] or [GDF5, GDF6, GDF7]. Interestingly, we note that a single linear ligand ordering, following the circle from BMP9 around to BMP10, was consistent with all observed equivalence maps, suggesting that ligand differences could fall on a linear continuum. While analysis of additional receptor contexts could further increase the number of global equivalence groups, this unified diagram efficiently summarizes the ligand interactions observed here.

A complementary approach to ligand classification is sequence similarity. Based on protein sequence, the ten ligands can be clustered into four groups: BMP2 and BMP4; BMP5, BMP6, and BMP7; BMP9 and BMP10; and GDF5, GDF6, and GDF7 (Figures 6C, S7A). Protein sequence similarity correlated more strongly with global equivalence relationships than with equivalence relationships in any single individual cell context (Figure 6D). In fact, the ‘global’ correlation coefficient, 0.80, approached the maximum value obtained with randomly sampled cell line combinations, 0.84 (Figure S7B). However, the lack of correlation with individual cell line data underscores how the canonical groupings of ligands based on sequence (Figure 6C) are not necessarily useful for predicting the response to a ligand combination or how the loss or addition of a BMP affects overall pathway responses.

Two outliers in the global correlation show how sequence is not a perfect predictor of even the global groups. First, [BMP5, BMP6, BMP7] had more similar pairwise interactions with [BMP9] than other BMPs with the same degree of sequence similarity. Conversely, [BMP9] and [BMP10] were the most dissimilar BMPs based on the equivalence groups, producing pairwise interactions of opposite sign, and yet were more similar by sequence to one another than to any other ligand. This suggests that the global equivalence groups contain functional information beyond what can be inferred from naïve comparisons of the full-length sequence. These could reflect, for example, effects of the 3D structures of specific ligand and receptor complexes. These results are generally consistent with previous results showing that mutation of a single amino acid or perturbation of sequences outside the receptor-binding domain can cause significant changes in BMP ligand behavior (Klammert et al., 2015; Seeherman et al., 2019).

### A mathematical model of competitive ligand-receptor interactions can explain combinatorial, context-dependent signaling

How could the context-dependent combinatorial interactions observed here emerge from underlying molecular interactions? The results above (Figure 6) demonstrate that receptor expression perturbations are sufficient to reprogram combinatorial responses. To understand how ligand-receptor interactions alone could give rise to the context-dependent pairwise responses observed here, we developed a mathematical model of BMP signaling that expresses pathway responses in terms of the formation and activity of ligand-receptor signaling complexes.

**Figure 7:**
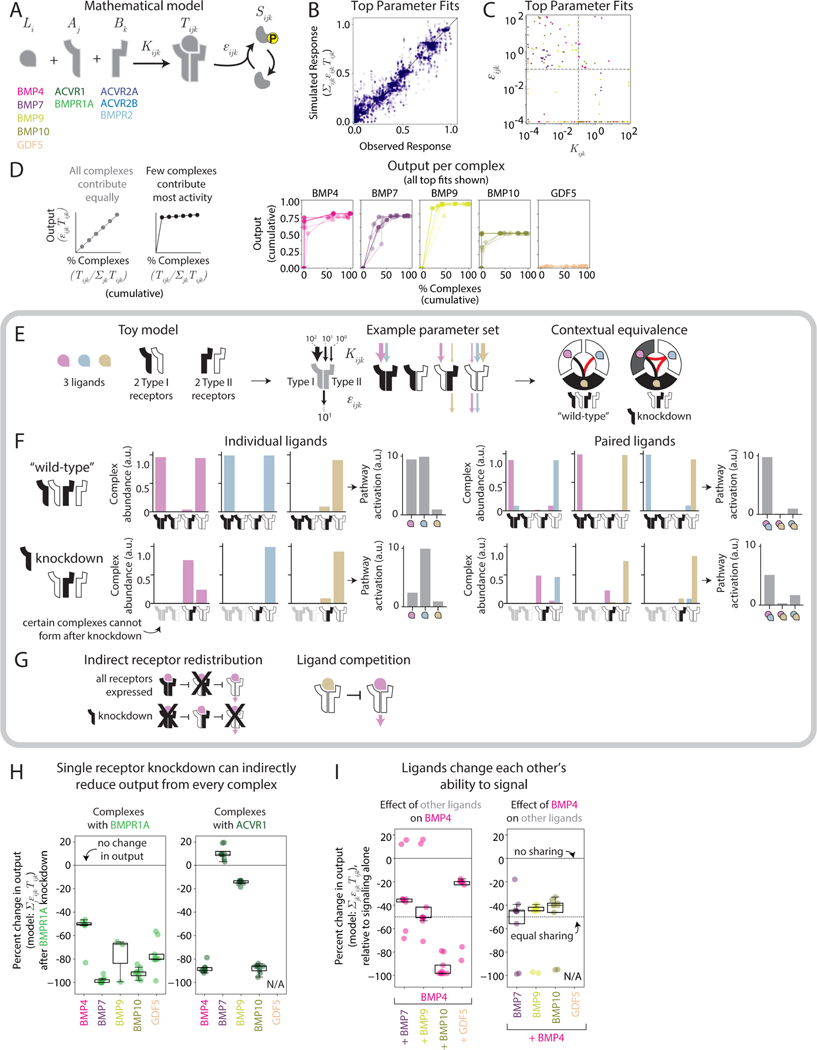
Mathematical model of receptor competition can explain contextual ligand equivalence groups A. In a simple BMP receptor competition model, mass action kinetics govern one-step assembly of ligand (*L*_*i*_), Type I receptor (*A*_*j*_), and Type II receptor (*B*_*k*_) into trimeric signaling complexes (*T*_*ijk*_) with some affinity (*K*_*ijk*_) and the activation of pathway output with some activity (*ε*_*ijk*_). The included components are listed beneath their associated variable. B. Observed responses, normalized between 0 and 1, correlate with simulated responses in all top parameter fits. Each point is one datapoint (720 total) fit by one parameter set (nine total). C. In all nine top parameter fits, the *K*_*ijk*_ values and *ε*_*ijk*_ values for each signaling complex tended to be anti-correlated (i.e. be respectively high and low, or vice versa). D. For individual ligands activating NMuMG, each complex’s inferred output complex is plotted against the cumulative percentage of the total complexes, with each line showing one of the top nine parameter fits. Complexes are sorted by increasing percentage. For BMP4 and BMP10 in particular, a minority of the complexes that form can produce the majority of the output. E. The toy BMP model contains only three ligands (pink, blue, gold); two Type I receptors (black, white); and two Type II receptors (black, white). The example parameter set of affinities and activities (used for (F) and (G)) is shown as arrow thicknesses, colored by ligand and adjacent to the relevant receptor dimer. This parameter set is sufficient to generate equivalence maps that vary with receptor expression. F. Solving the toy model with the example parameters from (E) in either a “wild-type” cell line (i.e. with all receptors expressed at a level of 1) or a “knockdown” cell line (with the removal of one receptor) revealed ligand-specific distributions of signaling complexes. Signaling complex abundance is shown for each receptor dimer, with the bar color showing which ligand is bound. Gray bar plots to the right of complex abundance plots show the summed pathway activation. Individual ligand strengths vary with knockdown, and pairwise interactions can also change. G. (*left*) The pink ligand can form three of the four possible receptor dimers and shows the effect of receptor knockdown on receptor distribution between dimers. When all receptors are expressed, the pink ligand’s strong binding to the black/black complex prohibits the formation of the white/black complex, allowing the formation of the low affinity white/white complex. By contrast, knockdown of the black Type I receptor allows the formation of the white/black complex, inhibiting the formation of the white/white complex, which is the most active. Thus, knockdown of a single receptor can reduce a ligand’s response via redistribution of unperturbed receptors. (*right*) Ligand competition similarly determines the distribution of high and low activity complexes, as competition between the gold and pink ligands can reassemble the white receptors into the lower activity complexes. H. The percent change in individual ligand output (relative to NMuMG) from BMPR1A-containing complexes (computed in the model) decreases when BMPR1A is knocked down, as expected. All other output must be produced via ACVR1, the remaining Type I receptor, but this output can also decrease following BMPR1A knockdown, due to the rearrangement of Type II receptors that shift ACVR1 to mostly inactive receptor complexes. All top parameter fits are plotted, unless the absolute change is small (i.e. <0.01 change in response). I. BMPs signaling in pairs compete for receptors and can produce more or less output as a result of this competition, as shown by the percent change (computed in the model) in BMP4 output when paired with other ligands (left) or in other ligands when paired with BMP4 (right) The model predicts that the additive interaction between BMP4 and BMP7 or BMP4 and BMP9 emerges from either equal sharing of receptors (i.e. each ligand produces 50% of the output produced when signaling individually) or BMP4 competing all receptors away from the other ligands. By contrast, the competition between BMP4 and BMP10 completely removes BMP4 signaling and reduces BMP10 signaling by half, producing an imbalance interaction. GDF5 can slightly antagonize BMP4, while BMP4 completely removes the small activation by GDF5. All top parameter fits are plotted, unless the absolute change is small (i.e. <0.01 change in response). See also Figures S8, S9.

The model assumes that each ligand variant, *L*_*i*_, can bind to any pair of Type I and Type II receptors, *A*_*j*_ and *B*_*k*_ respectively, with affinity *K*_*ijk*_ to produce a trimeric, ligand-receptor signaling complex, *T*_*ijk*_ (Figure 7A), whose binding is captured by a set of ordinary differential equations describing mass-action kinetics. Each signaling complex can then phosphorylate SMAD1/5/8 proteins with its own specific kinase activity *ε*_*ijk*_. We note that *ε*_*ijk*_ is an effective parameter that incorporates the combined effects of multiple molecular mechanisms through which receptor kinase activity can depend on the composition of a signaling complex (Nickel and Mueller, 2019; Ramachandran et al., 2021). Receptors are expressed at specific total levels, $A_{j}^{0}$ or $B_{j}^{0}$, which are assumed to not change over the course of the experiment, consistent with prior data (STAR Methods). The limited total amount of each receptor generates competition among ligands for available receptors, such that pathway activity depends on ligand combinations and concentrations (Antebi et al., 2017).

This model has several major simplifications compared to the actual BMP pathway. First, it neglects co-receptors, extracellular regulation by secreted inhibitors, inhibitory SMADs, and activity-dependent receptor regulation (Dutko et al., 2021; Feng and Derynck, 2005; Lucarelli et al., 2018; Miyazawa and Miyazono, 2017; Murakami et al., 2003; Ross and Hill, 2008). All these factors play important roles in the full BMP pathway and could impact ligand integration in natural contexts but were not perturbed in our experiments. Second, based on the relatively steady responses to BMP ligands over the timescales of the signaling response, the model neglects potential roles for dynamic feedback through inhibitory SMADs or activity-dependent receptor regulation (Antebi et al., 2017). Finally, for simplicity the model treats signaling complexes as comprised of just one Type I and one Type II receptor, rather than tetramers containing two receptors of each type. However, it could be generalized in a straightforward way to allow tetrameric receptors, as may be necessary to explain recent results with ACVR1 (Little and Mullins, 2009). Nonetheless, although highly simplified, this model is sufficient to account for the ligand integration behaviors observed here and in related studies (Antebi et al., 2017; Su et al., 2020).

We asked whether this model could be sufficient to explain the complex set of observed equivalence groups. To further simplify the analysis, we only included one ligand from each global equivalence group and restricted the model to the five receptors expressed in NMuMG cells, which are also the most broadly expressed in other cell contexts: the Type I receptors ACVR1 and BMPR1A and the Type II receptors ACVR2A, ACVR2B, and BMPR2 (Figures 7A, S4A, S5A). We then performed 6000 independent parameter fits to the data and identified 9 parameter sets that matched individual and pairwise interactions (Figures 7B, S8A; STAR Methods). The lack of a single unique best fit was in part due to weak signaling ligands, such as GDF5, whose weakness could arise either from low affinity or activity (or both). In the future, additional data obtained in cell contexts where such ligands do activate could help to further constrain these parameters. Nonetheless, the best fitting parameter sets shared similar values for many individual parameters and provided insight into how complex equivalence relationships can emerge from competitive ligand-receptor interactions (Figures S8B-O).

Inspection of the best fit parameter sets revealed two patterns in the parameter values themselves. First, all ligands were promiscuous, with substantial but variable affinities for most or all receptors. BMP7 and GDF5 had lower affinities overall across all complexes, while BMP4, BMP9, and BMP10 had higher affinities for select complexes (Figures S8D-I, S9A). Consequently, each ligand exhibited a unique distribution of signaling complexes in any given cell line (e.g., Figure S9B). Second, many signaling complexes exhibited strong affinity but weak activity, or vice versa (Figures 7C, S9A). As a result, in the model, most of the signal was typically generated by a small fraction of signaling complexes (Figures 7D, S9B,C). Together, these parameter fits indicate that ligand-receptor interactions alone could be sufficient to generate the observed repertoire of combinatorial, context-dependent behaviors.

### In the model, context-dependence emerges from redistribution of signaling complexes

In the top model fits, context-dependent ligand interactions result from two features of the model. First, due to competitive, mass action kinetics, perturbation of a single receptor or ligand concentration can redistribute the other ligands and receptors present into new signaling complexes. Second, because each signaling complex has a distinct activity, *ε_ijk_*, this redistribution can increase or decrease the total amount of signaling. Together, these features can provide an explanation of changing responses with differences in receptor or ligand context. To better understand these features, we first analyzed an even simpler “toy” version, consisting of only three ligands, two Type I and two Type II receptor variants (Figure 7E-F) with a hypothetical parameter set (Figure 7E) we constructed to qualitatively recapitulate non-additive ligand interactions and receptor context-dependence (Figure 7F).

The toy model shows how receptor redistribution can generate complex equivalence groups. For example, one ligand (pink) exhibits strong, intermediate, and low (but non-zero) propensities to form three different complexes of two Type I and two Type II receptors, with the complex with the lowest propensity to form producing the most output (i.e., highest *ε*_*ijk*_; Figure 7E, pink arrows), mirroring with anti-correlated affinity and activity observed in some best fit parameter sets. When all receptors are expressed at equal levels (“wild-type” context), the high affinity (black/black) complex forms efficiently, titrating away the black Type II receptor subunits required to form the intermediate affinity (white/black) complex (Figure 7F, upper left panel, and Figure 7G). This leaves the white receptor subunits free to form lower affinity (white/white) complexes. Thus, in this example, one can observe that the abundance of each signaling complex depends on its own affinity, the affinities of competing complexes, and the total expression levels of all receptor subunits.

In this background, perturbing the expression of a single receptor subunit can either increase or decrease the abundances of various complexes (Figure 7F, lower left panel). For instance, decreasing expression of the black Type I receptor prevents formation of the high affinity (black/black) receptor. This frees Type II receptors from that complex to form the intermediate affinity (white/black) complex, which in turn reduces formation of the low affinity (white/white) complex (Figure 7G). In this example, the knockdown directly inhibits the formation of the non-signaling black/black complex, but receptor rearrangements further decrease the formation of the signaling white/white complex. Consequently, knockdown of the black receptor decreases the activity of the pink ligand through a distinct, non-perturbed receptor. Thus, perturbing a single receptor can generate a chain of direct and indirect effects on signaling complexes that do or do not contain it.

Turning to the full model, we found that the top parameter fits exhibited similar effects in response to receptor perturbations. For example, knockdown of BMPR1A directly diminished signaling by BMP4 and BMP10 through BMPR1A itself, as expected (Figure 7H). However, it also diminished signaling by the same ligands through ACVR1, the only other expressed Type I receptor (Figures 7H, S9D,E). This effect resulted indirectly from the reduced availability of BMPR1A, which released ACVR2B subunits to complex with ACVR1 instead of BMPR1A (Figure S9D,E). As a result, BMP4 and BMP10 preferentially form weaker signaling complexes with ACVR1 and ACVR2B. In this case, knockdown of one receptor can directly decrease output through its own complexes, while indirectly affecting output through other receptor complexes.

The effect of adding one ligand to another (ligand context) can also be understood in terms of redistribution of signaling complexes. In the toy model, the gold ligand has high and low affinities for the white/white and white/black receptors, respectively (Figure 7E, parameter set). As a result, it can outcompete the pink ligand for binding with the white/white receptor complex, displacing the pink ligand to bind lower affinity receptors (Figure 7F, “paired ligands”). Since the pink ligand requires the white/white complex for signaling, while the gold ligand does not signal through it all, the gold ligand can effectively inhibit activation by the pink ligand (Figure 7G).

A similar ligand redistribution effect appears in the top fits to the experimental data. For example, in NMuMG cells, we observed that adding BMP10 to BMP4 reduces pathway activation below that produced by either ligand alone (suppressive interaction, Figure 3E). In the model fits, this occurs because the addition of BMP10 redistributes BMP4 to predominantly form lower activity signaling complexes (Figures 7I, S9F). By contrast, addition of BMP7 to BMP4 also redistributed signaling complexes, but did so without producing a net effect on total activity (Figures 7I, S9F). In the majority of parameter fits, combining BMP4 and BMP7 reduces signaling by each ligand by half, such that the total output of both ligands matches that produced by each ligand alone (Figures 7I, S9F). In other parameter fits, BMP4 or BMP7 competes away most receptors from the other ligand, such that it produces the same output individually as in the pair, also consistent with a saturated additive response (Figure 7I). Thus, addition of one ligand can redistribute other ligands to different signaling complexes to generate non-additive, but also additive, effects.

The combination of ligand and receptor redistribution can explain how pairwise interactions vary among cell types. In the toy model, we introduced a third blue ligand whose receptor preferences and activity profile mostly overlap with those of the pink ligand. A key difference, however, is that the pink, but not the blue, ligand can form an additional non-signaling complex with the white/black receptor pair. When all receptors are present, blue and pink ligands presented individually form a similar distribution of signaling complexes (Figure 7F, upper left panel). When presented together, affinity preferences segregate the two ligands to distinct receptors, but preserve total activity (Figure 7F, upper right panel). However, following knockdown of the black receptor, the pink ligand, but not the blue ligand, can be sequestered in an inactive complex with the white/black receptor, lowering its individual signaling strength (Figure 7F, lower left panel). Finally, when combined, the inactive pink/white/black complex reduces the availability of white Type I receptors, limiting signaling by the blue ligand (Figure 7F, lower right panel). As a result, pink and blue ligands can combine additively in the original cell context, but antagonistically in the knockdown. Taken together, these results show that the relatively simple principle of redistribution of ligands and receptors into signaling complexes with varying activity can explain the combinatorial and contextual nature of BMP signaling observed here. However, additional experiments will be necessary to fully determine the underlying biophysical mechanisms.

## Discussion

BMP signaling is both combinatorial and contextual. The effect of one ligand on signaling depends, in general, on which other ligands are also present and which cell type they are activating. Individual BMP ligands are known to preferentially bind and signal through specific receptors. However, receptor preferences cannot by themselves explain these two types of effects. Here, we introduced contextual ligand equivalence groups as an alternative framework to classify ligands based on how they signal in combinations, across different cellular contexts. We showed how systematic pairwise measurements define equivalence groups, each comprising a set of ligands that interact similarly with all other ligands when activating a given cell type (Figure 3F). These equivalence groups are contextual, depending on the receptor expression profile of the target cell (Figures 4–6). In contrast to ligand classifications based on biochemical properties like affinity, contextual equivalence groups classify BMP ligands and receptors based on their emergent functional properties.

This analysis confirms known features of specific ligand-receptor interactions and reveals new ones. For example, previous work has classified ligands into approximately four groups based on overall sequence homology (Kopf et al., 2014). Here, clustering ligands by the similarity of their pairwise interactions across contexts reproduced three of these groups: [BMP2, BMP4], [BMP5, BMP6, BMP7], and [GDF5, GDF6, GDF7], but revealed striking functional differences between the members of the fourth group, BMP9 and BMP10 (Figure 6B). BMP9 and BMP10 were in fact the most functionally dissimilar ligands among the set of 10 (Figure 6D). All synergistic interactions involved BMP9, while all suppressive interactions involved BMP10. In addition to delineating groups, the approach here also revealed functional differences between groups. For example, we found that the ligands [BMP5, BMP6, BMP7] are strong activators and rarely susceptible to receptor perturbation or antagonism by other ligands. By contrast, the equally strong activating ligands [BMP2, BMP4] were susceptible to antagonism by other ligands (Figure 6B).

Our results underscore the importance of classifying ligands with respect to particular cell contexts. For example, [BMP5, BMP6, BMP7] and [BMP9] function equivalently in many of the cell contexts analyzed here despite representing distinct global equivalence groups (Figures 6A,B). Similarly 8 of the 10 ligands, representing multiple global equivalence groups, functioned equivalently when BMPR1B was ectopically expressed (Figure 6A). In cases like this, where the new cell context involves ectopic expression of a single receptor, the merging of distinct equivalence groups may reflect relief of competition for otherwise limiting receptors, resulting in an increase in additive interactions. For example, BMP10’s suppressive and antagonistic interactions in the NMuMG context were removed following ectopic expression of ACVRL1, a receptor it binds with high affinity and strongly activates (David et al., 2007). In another case, antagonism between GDFs and other ligands was reversed following ectopic expression of BMPR1B, a preferred receptor of the GDF ligands (Nishitoh et al., 1996). Overall, these changes in ligand equivalence show that information about receptor expression is crucial for understanding and ultimately predicting ligand responses in a new cell context.

Contextual equivalence groups could help explain counterintuitive results in developmental genetics. For example, BMP10 signals in both developing heart and vasculature (Chen et al., 2013). Although BMP9 has the closest sequence similarity to BMP10, the contextual equivalence maps show that BMP9 can only substitute for BMP10 in the presence of ACVRL1 (Figures 6A, S6D). Consistent with this picture, BMP9 rescued loss of BMP10 in developing mouse vasculature, which expresses ACVRL1, but not in heart, which does not (Chen et al., 2013; Seki et al., 2003). Another example of receptor context occurs in the developing joint interzone, where BMP2 and GDF5 are broadly expressed (Lyons and Rosen, 2019). In the absence of BMPR1B, these ligands antagonize each other, which may explain why they suppress chondrogenesis in interzone cell types expressing BMPR1A, while promoting chondrogenesis in specific BMPR1Bexpressing cartilage progenitors.

Equivalence groups also reveal the effects of ligand context. For example, in cell lines where BMP4 and BMP7 are strong activators, they can only replace each other in the absence of antagonistic ligands such as GDF5, GDF6, GDF7, or BMP10 (Figure 6A, “NMuMG,” “ACVRL1 EE,” “BMPR1B EE”). Consistent with this, BMP4 and BMP7 functioned redundantly in the developing kidney (Oxburgh et al., 2005), where those antagonizing ligands are not expressed (Godin et al., 1999). By contrast, loss of BMP4 produced more pronounced effects than loss of BMP7 in the context of developing skeleton with conditional BMP2 knockout (Bandyopadhyay et al., 2006), a context where GDF5 is expressed and plays a key role (Storm and Kingsley, 1999).

In addition to mapping the context-dependent combinatorial interactions of BMP ligands, we sought to understand how these effects could arise. Although context-dependence could emerge at many levels in BMP signaling (Massagué, 2012), the strong effect of single receptor perturbations suggested that ligand-receptor interactions likely play a key role in generating the responses observed here. In fact, a simple model of ligand-receptor interactions is consistent with the data. Fitting the model to these data further identified sets of effective ligand affinity and activity values sufficient to generate the full set of context-dependent pairwise responses. Interestingly, top parameter fits often showed inverse relationships between activity and affinity. A parallel computational study of the BMP pathway showed that such anti-correlations facilitated combinatorial addressing, in which different ligand combinations can selectively activate distinct cell types (Su et al., 2020). Nevertheless, the model is currently underdetermined and does not rule out other models incorporating more or different pathway interactions. Additional experiments will be necessary to better constrain pathway parameters in this model and compare it to alternatives.

While it is difficult to relate published affinities of BMP ligands for individual receptor subunits to the *K*_*ijk*_ parameters that describe trimeric complex formation in this simple model, key aspects of this model are consistent with previously observed features of BMP signaling. For example, loss of BMP or related TGF-β receptors was shown to indirectly affect the abundance of other receptor complexes (Hiepen et al., 2019; Lowery et al., 2015; Razavi et al., 2019), consistent with the receptor redistributions predicted by the model (Figure 7H). In addition, recent work in zebrafish showed that the kinase activity of the BMPR1A homolog, but not ACVR1, was dispensable for BMP-driven dorsoventral axis formation (Dutko et al., 2021). This result is broadly consistent with the model fitting results, in which ACVR1 receptors were responsible for most signal output (Figures 7D and S9C,E). Finally, in previous work, BMPR2 knockdown produced unexpected and ligand-specific effects across a variety of cell types (Han et al., 2013; Hiepen et al., 2019; Olsen et al., 2018; Theilmann et al., 2020; Yu et al., 2005). For example, in pulmonary artery cells and myeloma cells, BMPR2 knockdown either did not affect or decreased signaling by BMP4 and BMP10, but increased or had no effect, respectively, on signaling by BMP7 and BMP9. Consistent with these results, BMP7 and BMP9 could bind and activate a variety of receptor dimers in the parameter fits, whereas BMP4 and BMP10 were more dependent on BMPR2 (Figure S8D-I).

The contextual equivalence analysis of BMP signaling introduced here can be improved and extended in several ways. First, analyzing ligand equivalence in additional receptor contexts would provide more complete coverage of possible BMP signaling contexts. Second, including heterodimeric ligands such as BMP2/7 could reveal whether these ligands have distinct contextual properties from their related homodimers, in addition to their unique individual properties (Valera et al., 2010). Third, many natural contexts use three or more ligands simultaneously. The approach taken here could be extended to higher order ligand combinations to determine whether pairwise interactions completely determine multi-ligand effects or whether higher-order interactions, possibly due to receptor clustering, are important (Ramachandran et al., 2021; Schneidman et al., 2006). In this way, it should be possible to construct equivalence maps for many of the most important cell contexts, allowing researchers to ‘look up’ expected combinatorial interactions, explain complex phenomena, and even predict cellular responses in their systems of interest.

Finally, we emphasize that ligand classification by pairwise interactions can be further generalized in several ways. First, the signaling of BMP heterodimers could be studied with the same SMAD1/5/8 reporters and modeled as ligands with distinct affinities and activities, due to their novel Type I receptor binding sites (Dutko et al., 2021). Second, additional TGF-β family ligands, such as TGF-β or activin, could be included. These ligands regulate phosphorylation of the related SMAD2/3 transcription factors, share some but not all receptors with BMP ligands (Razavi et al., 2019), and exhibit distinct activation dynamics that may allow more complex ligand integration (Weiss and Attisano, 2013; Zi et al., 2012). Incorporating this related set of ligands could reveal more complex equivalence relationships and provide deeper insight into a critical signaling system in cancer and wound healing (Bierie and Moses, 2010; Pakyari et al., 2013). Third, analyzing a broader set of BMP targets, including non-canonical outputs and broad repertoires of target genes, could determine whether different outputs follow the same ligand equivalence relationships as SMAD1/5/8 phosphorylation (Blitz and Cho, 2009; Gunnell et al., 2010; Saxena et al., 2018). Fourth, a similar approach could be applied to other pathways, such as Wnt or FGF, which also exhibit promiscuous interactions among multiple ligand and receptor variants (Gleason et al., 2006; Kettunen and Thesleff, 1998). In the longer term, equivalence groups could function as a set of ‘periodic tables’ that organize ligands according to their functional activity and contextual interactions in a unified manner. Such a classification could help us understand why specific components operate together in different developmental contexts and allow context-specific control of pathway activity for therapeutic applications.

## STAR Methods

### Resource Availability

#### Lead Contact

Further information and requests for resources and reagents should be directed to and will be fulfilled by the Lead Contact, Michael Elowitz (melowitz@caltech.edu).

#### Materials Availability

Plasmids and cell lines generated for this paper, as indicated in the Key Resources Table, are available from the Lead Contact upon request. The recombinant proteins, lentiviral particles, siRNAs, and qPCR probes used in this paper are described in Tables S1-S4 and can be purchased from commercial sources, as detailed in the Key Resources Table.

#### Data and Code availability

- **Source data statement:** Flow cytometry and qPCR data have been deposited at Caltech DATA and are publicly available as of the date of publication. DOIs are listed in the Key Resources Table. This paper also analyzes existing, publicly available data. The accession numbers for these datasets are also listed in the Key Resources Table.
- **Code statement:** All original code has been deposited at Caltech DATA and is publicly available as of the date of publication. DOIs are listed in the key resources table.
- Any additional information required to reanalyze the data reported in this paper is available from the lead contact upon request.

### Experimental Model and Subject Details

#### Tissue culture and cell lines

NMuMG (NAMRU Mouse Mammary Gland cells, female) were acquired from ATCC (CRL-1636). E14 cells (mouse embryonic stem cells (mESCs), E14Tg2a.4, male) were obtained from Bill Skarnes and Peri Tate. All cells were cultured in a humidity-controlled chamber at 37°C with 5% CO2. NMuMG cells were cultured in DMEM supplemented with 10% FBS (VWR #311K18), 1mM sodium pyruvate, 1unit/mL penicillin, 1μg/mL streptomycin, 2mM L-glutamine, and 1X MEM non-essential amino acids. mESCs were plated on tissue culture plates pre-coated with 0.1% gelatin and cultured in standard pluripotency-maintaining conditions (Smith, 2001) using DMEM supplemented with 15% FBS (ES qualified, GIBCO #16141), 1mM sodium pyruvate, 1unit/mL penicillin, 1μg/mL streptomycin, 2mM L-glutamine, 1X MEM non-essential amino acids, 55μM β-mercaptoethanol, and 1000units/mL leukemia inhibitory factor (LIF).

#### Reporter cell line construction

To construct reporter cell lines, a plasmid harboring the BMP response element (Korchynskyi and ten Dijke, 2002) in the enhancer region of a Major Late Promoter (MLP) driving the expression of an H2B-mCitrine protein fusion was integrated into the NMuMG genome using PiggyBac integration (System Biosciences). After transfection, cells were selected with 100μg/mL hygromycin. The base NMuMG reporter was a clonal cell line generated by limiting dilution. This reporter construct exhibited lower background YFP expression and increased signaling dynamic range relative to a similar NMuMG transcriptional reporter (Antebi et al., 2017), consistent with other observations of MLP’s lower background relative to mCMV (Ede et al., 2016). A previously-reported mESC reporter was used (Antebi et al., 2017). To construct this reporter, a related construct, carrying minimal CMV in place of MLP, was randomly integrated into the E14 genome using the FugeneHD reagent and a clonal population selected by colony picking.

Because of the known direct physical interactions between Smad1/5/8 and BMP Type I receptors (Figure 1A), as well as correlations with measurements collected on timescales too short for transcriptional feedback, we analyze our YFP measurements with the simplifying assumption that the measured YFP reflects the integrated, total pool of activated Smad/1/58 and is largely determined by receptor-ligand interactions, with only smaller contributions from downstream components.

#### Recombinant BMP ligands

Recombinant BMP ligands were ordered from R&D systems, as indicated in the Key Resources Table and Table S1. R&D ensures the purity of their reagents with a proprietary purification process, SDS-PAGE to verify the molecular weight of the purified product, and N-terminal sequencing to verify the sequence. They further verify the activity of purified protein by activation of alkaline phosphatase and require that the ED50 of activation fall into a specified absolute range and within 2-fold of all other lots. Given the large dynamic range of these ligands (a minimum of 10-fold), a 2-fold difference in activity per mass would not significantly affect the results presented here, though we note that the largest variability in our results emerges at intermediate concentrations, where this lot-to-lot variability could have the largest effect. Previous work studying pairwise interactions between BMPs produced the same pairwise interactions, though at slightly different masses, when comparing ligands purified by R&D and another supplier (Antebi et al., 2017).

### Method Details

#### Quantifying BMP responses with flow cytometry

To assay BMP pathway activity, we genomically integrated a transcriptional reporter into all cell lines as described above (see “Reporter cell line construction”), which expressed YFP proportional to pSMAD activation (Antebi et al., 2017). To measure how a given ligand or combination of ligands activated the pathway, we used the following protocol in all robotic or manual experiments. Cells were plated at a confluency that, assuming a 24 hour doubling time, would produce sufficient cells for flow cytometry at the experiment endpoint (i.e. at least 5000 cells 36 hours after plating in a 96 well format, or about 20–30% confluent at the start of the experiment). Twelve hours after plating, cell media was removed and replaced with ligand-containing media. Twenty-four hours after ligands were added, cells were lifted from the plate for flow cytometry, by five minute incubation at 37°C in 0.25% Trypsin (or Accutase, for mESCs) following PBS wash. Trypsinization was quenched by resuspending cells in flow buffer, (HBSS containing 2.5mg/mL Bovine Serum Albumin (BSA)). Cells were then filtered through a 40μm mesh and analyzed by flow cytometry (MACSQuant VYB, Miltenyi or CytoFLEX, Beckman Coulter). Because of cell-line specific difficulties with cellular aggregation following filtering, alternative flow buffer formulations additionally included either 1mM ethylenediaminetetraacetic acid (EDTA) or 200U/mL DNAse I in some cases.

#### Robotic liquid-handling protocol

For each cell line, a similar protocol was used to generate a full pairwise dataset. First, the transcriptional BMP reporter cell line was generated, and the receptor profile was assayed by qPCR. The only exception was the mESC reporter, whose receptor profile was obtained from a prior publication that assayed receptor expression by RNA-Seq (Antebi et al., 2017). Then, we determined the dynamic range of each ligand in this new receptor context. To do this, we completed at least one biological replicate of single ligand dose responses up to very high concentrations that were expected to approach saturation (e.g. 3μg/mL), with a high fold dilution to capture the full dynamic range. These dose responses fell into three types: saturating activators, non-saturating activators, and non-activators. For saturating activators, we selected a maximum concentration and fold dilution that sampled concentrations corresponding roughly to 25, 50, 75, and 100% of maximal activation. For non-saturating activators, maximal activation was unknown. To avoid supraphysiological concentrations, we limited the maximum concentration to 2 or 3μg/mL and used a minimum 2-fold dilution to sample whatever of the dynamic range was accessible. For non-activators, there was no dynamic range. We therefore selected maximum concentrations and fold dilutions that approximated the median dynamic range of the activating ligands, reasoning that these highly homologous ligands had, to a first approximation, reasonably similar concentrations over which they were most active. The full list of all concentrations and fold dilutions used for each cell line is in Table S1.

Next, we assayed all pairwise interactions, with single ligand gradients as well as “pseudo-pairs” of ligands with themselves as controls. For ten ligands, the screen included 10 gradients, 10 “psuedo-pairs,” and the 45 possible pairs, or 65 rows of measurements. Each row included a no BMP control, plus the nine ratios at which each pair (ligand by itself, ligand paired with itself, or ligand paired with another ligand) was assessed. The order of the rows and of the ligands was randomized between biological repeats, and at least three biological repeats were completed for each cell line.

To expose cells to these complex ligand combinations, we used a custom liquid-handling protocol on the Tecan EVO Freedom 200 base unit, which was housed in a sterile, laminar flow hood. To start the experiment, we manually plated cells eight to twelve hours before the start of the robotic protocol and prepared serial dilutions of the ten ligands by hand. The robotic protocol then added the precise ligand combinations to the appropriate plates, and timed the start of each plate to match the staggered analysis of each plate twenty-four hours after the ligand combinations were added. Specifically, at the appropriate time point, the robot removed a plate from the incubator, aspirated the media, replaced it with the appropriate ligand-containing media, and returned cells to the incubator. The robot incubator was fed 5% CO_2_. All plates were then collected from the robot incubator at the end of the experiment.

Finally, we quantified cell responses in each of these ligand conditions. 20 to 24 hours after the BMP ligands were added, we measured YFP of each well by flow cytometry, as described above (see “Quantifying BMP reporter with flow cytometry”). We analyzed the resulting flow data with custom MATLAB scripts available at https://doi.org/10.22002/D1.1693, following the steps described below in “Data Processing.”

#### Knockdown and ectopic expression of receptors

Individual BMP receptors were knocked down or ectopically expressed in the base NMuMG reporter cell line. For receptor knockdown, lentiviral particles containing constructs for constitutive shRNA expression reported by mCherry with a puromycin-resistance gene (SMARTvector, Dharmacon) were transduced into cells. Per manufacturer’s instructions for determining optimal transduction conditions, we transduced cells at an MOI of 20, using serum-free NMuMG growth media supplemented with 10μg/mL polybrene. 48h after transduction, cells were selected and continuously maintained in 3μg/mL puromycin. Cells with BMPR1A and BMPR2 knockdowns were polyclonal populations generated by a single shRNA. Cells with ACVR1 knockdown were a clonal population selected by limiting dilution of cells transduced with a pool of three shRNAs. For a list of the shRNAs used, see Table S2.

For ectopic expression of BMP receptors, constructs for constitutive expression of mouse receptor cDNAs, reported by mTurquoise and co-expressed with a geneticin-resistance gene, were integrated into the base NMuMG reporter by PiggyBac integration (Systems Bioscience), using previously-reported plasmids (Antebi et al., 2017). Cells were selected and maintained with 500μg/mL geneticin.

#### Transient siRNA knockdown

Cells were plated at 40% confluency in a 24 well plate format with 30μM total siRNA (ThermoFisher Silencer Select #4390771) and 3μL RNAiMAX (Life Technologies). For every gene, a pool of two distinct siRNAs was used, listed in Table S3. Cells were passaged after 24 hr and then used for the relevant experiments.

#### RT-qPCR

Total RNA was harvested from cell lysate using the RNeasy mini kit (QIAGEN), and cDNA was generated from 1μg of RNA using the iScript cDNA synthesis kit (BioRad) per the manufacturer’s instructions. Primers and probes for specific genes (Table S4) were purchased from IDT. Reactions were performed using 1:40 dilution of the cDNA synthesis product with either IQ SYBR Green Supermix or SsoAdvanced Universal probes Supermix (BioRad). Cycling was carried out on a BioRad CFX96 thermocycler using an initial denaturing incubation of 95°C for 3 min followed by 39 cycles of (95°C for 15s, followed by 60°C for 30s). Each reaction was run at least in duplicate.

### Quantification and Statistical Analysis

#### Analysis of RNA-Seq datasets

To survey BMP ligand and receptor expression across various tissues, we selected relevant datasets from a collection of bulk, mouse RNA-Seq datasets (Lachmann et al., 2018). Specifically, datasets that indicated the sample library contained transcriptomic cDNA prepared from polyA RNA and that indicated a tissue-specific source were kept. RNA-Seq quantification of BMP receptor expression in the NMuMG parental cell line and the mESC reporter cell line (cf. Figure 4A) was taken from GSE98674 (Antebi et al., 2017). We analyzed these data with custom MATLAB and Python scripts available at https://doi.org/10.22002/D1.1693, where the code is ordered by figure panel.

#### BMP alignment and clustering

To analyze amino acid sequences of BMP ligands, the relevant recombinant sequences (indicated by R&D Biosystems) were globally aligned with MATLAB’s “multialign” function, using default parameters. Distance between sequences was computed by the BLOSUM50 matrix, comparing only positions with no gaps across all aligned ligands, and they were clustered with average-distance linkage. We analyzed these data with custom MATLAB and Python scripts available at https://doi.org/10.22002/D1.1693, where the code is ordered by figure panel.

#### Data processing

All flow cytometry data for gradients and rims was gathered by the same method, implemented manually or robotically (see “Quantifying BMP reporter” and “Robotic liquid-handling protocol” above). The resulting flow data were analyzed in the following manner. Using a custom MATLAB GUI (https://antebilab.github.io/easyflow/), cells were gated on Forward Scatter (FSC) and Side Scatter (SSC) to exclude doublets and cell debris. Median YFP used to summarize pathway activation in each well. A custom MATLAB script (available at https://doi.org/10.22002/D1.1693) then dropped any wells with fewer than the threshold number of cells (i.e. 500), reordered wells to match standard ligand order, and subtracted background. For rims, an additional step minimized noise between the eleven plates, which, due to small variation in plate preparation and processing times, had incubation times varying from 20 to 24 hours. Assuming linear accumulation of YFP with time, variation in incubation generates a fold change in background-subtracted YFP. Therefore, each plate was linearly rescaled to a reference plate to minimize total least squares error between technical replicates shared between the two plates. Following standardization of each biological replicate, they were aggregated into a cell-line specific dataset. To reduce variance due to biological noise, each biological replicate was rescaled by a single linear factor that minimized total least squares error between all corresponding measurements in the two replicates.

To complete gradient analysis, all biological replicates were fit to a Hill function by minimizing total least squares error between the Hill fit and all rescaled biological replicates. To complete rim analysis, Relative Ligand Strength (RLS) and Interaction Coefficients (IC) were computed. RLS is the ratio of each ligand’s saturating activation strength to the strongest ligand’s saturating activation strength. However, the IC metric required more careful analysis to ensure robustness to technical and biological noise, as described in the following section.

#### Interaction Coefficient

##### BMP ligand synergy distinct from drug synergy

Classification of synergy and antagonism occurs in many fields, including the study of drug interactions and gene epistasis (Costanzo et al., 2016; Meyer et al., 2019; Russ and Kishony, 2018; Yeh et al., 2006; Zimmer et al., 2016). However, these metrics do not have clear analogies for classifying ligand interactions for at least two reasons.

First, all classifications of pairwise interactions, when agnostic of mechanism, depend on a phenotypic definition of additivity, i.e. a non-interacting relationship. However, the many definitions of additivity developed for drug interactions are not consistent with each other and not necessarily appropriate to describe ligands activating the same pathway. As an example of this, most definitions of additivity descend from approaches defined by Loewe (Loewe, 1928) and Bliss (Bliss, 1939), who predicted that additive outcomes would be the sum of each component’s dose or effect, respectively (Russ and Kishony, 2018). However, these assumptions make little sense in the context of two signaling proteins that use the same signaling components to produce their individual effects; indeed, individually saturating concentrations are not expected to sum in their effects or their normalized dose. A third approach to additivity considers the effect of each drug as an independent event, such that a non-additive response is identified as a deviation from the known joint probability of the independent outcomes (Yeh et al., 2006). But the sharing of pathway components between the two ligands makes this assumption of independence inappropriate.

Second, classifications of interaction type depend on well-defined regimes of behavior. The effect of drug combinations on growth rate has distinct boundaries for these regimes, but there are not clear boundaries for pathway activation. For example, the largest effect of a drug is to decrease growth rate to zero, but the upper bound of a signaling protein’s effect could be set by any one of the pathway components being limiting and so depends heavily on cell context. Similarly, drugs can exhibit a “strong” form of antagonism when a combination causes cells to grow faster than in the absence of drugs. By contrast, signaling proteins cannot, in combination, decrease pathway activation below zero, i.e. the response in the absence of signaling proteins. Thus pathway activation requires a different separation between weak and strong antagonism.

##### An interaction metric specific to ligands

To address the incongruency of drug and ligand interactions, we developed the Interaction Coefficient. The goal of defining this metric was to quantify and compare specific ligand behaviors of interest, rather than providing a generic or alternative synergy metric to an increasingly large list (Russ and Kishony, 2018; Tekin et al., 2016; Vlot et al., 2019). Rather than presenting evidence for additivity or deviations from it, the IC separates qualitatively distinct pairwise response types. This is because ligand equivalence depends solely on the similarity of pairwise response types, regardless whether those types could be called additive or any other name.

To define the IC, we consider three boundaries between four qualitatively distinct pairwise response types. These boundaries are (1) the weaker individual ligand response, (2) the stronger individual response, and (3) their sum. Thus, the four regimes (cf. Figure S2A) correspond to a combinatorial response that is lower than both the individual responses (suppression), a response that interpolates the two individual responses (antagonism), a response that falls in between fully saturated and linearly additive (additive), and a response that is larger than the summed individual responses (synergy). Within each of these regimes, the strength of that behavior type can be computed by normalizing the observed response to the maximal effect size. This generates the following definition of IC:

$$IC=\frac{f(A+B)}{f(A)+f(B)},\;if\;f(A+B)\geq f(A)+f(B)\;IC=\frac{f(A+B)-f(B)}{\left[f(A)+f(B)]-f(B\right)},\;if\;f(A)+f(B)>f(A+B)>f(B)\;IC=\frac{f(A+B)-f(B)}{f(B)-f(A)},\;if\;f(B)\geq f(A)+f(B)>f(A)\;IC=\frac{f(A+B)}{f(A)}-2,\;if\;f(A)+f(B)\geq f(A+B)$$

where *f(X)* is the nonnegative pathway response to ligand input X, and *f(B) > f(A)*. Note that IC has no upper bound, consistent with the lack of upper bound on pathway activation, and that this calculation assumes all data have been background subtracted.

However, this definition of IC can misclassify small absolute changes in *f(A+B)* as strong pairwise interactions in a few cases. Specifically, if f(A)0 or f(B)f(A), the denominator of IC diverges, respectively, in the additive and antagonistic regimes, due to the effective collapse of one of the behavior regimes.

We therefore defined a discontinuous expression for IC, which normalizes each regime to a more intuitive standard that does not approach zero, which is the response to the stronger ligand individually, or *f(B)*. The Interaction Coefficient (IC) as reported in this paper is computed with the following piecewise formula:

$$IC=\frac{f(A+B)}{f(A)+f(B)},\;if\;f(A+B)\geq f(A)+f(B)\;IC=\frac{f(A+B)-f(B)}{f(B)}=\frac{f(A+B)}{f(B)}-1,\;if\;f(A)+f(B)>f(A+B)>f(B)\;IC=\frac{f(A+B)-f(B)}{f(B)}=\frac{f(A+B)}{f(B)}-1,\;if\;f(B)\geq f(A)+f(B)>f(A)\;IC=\frac{f(A+B)}{f(A)}-2,\;if\;f(A)\geq f(A+B)$$

where *f(X)* is the nonnegative pathway response to ligand input X, and *f(B) > f(A)*, as above. To describe the interaction of any ligand pair, the largest magnitude IC value, positive or negative, for all ratios of A to B is reported.

##### Quantifying interactions from noisy data

Because *f(X)* is sampled only three or four times from a broad, underlying distribution (cf. Figure S2B), we sought a robust metric to determine if two sets of measurements, *f(X)* and *f(Y)*, could be reasonably considered different from each other, in both a statistical and biological sense. Our first criterion was that the range of replicates of *f(X)* must not overlap with the range of *f(Y)* replicates. This is a more stringent requirement than more traditional measures of statistical differences, such as a *p*-value or computing the standard deviation of the two groups, which cannot be reliably computed from such a small number of replicates. Requiring non-overlap is roughly approximate to requiring that the effect size be larger than the experimental noise for that measurement.

We found that even technical replicates collected on different days (i.e. *f(X)* measured in triplicate on different days) were sometimes non-overlapping, indicating these measurements were distributed over a large range. Therefore, we added an additional criterion that the degree of non-overlap must exceed a certain threshold. Many of the apparently spurious classifications occurred for low YFP values that were a large percentage change in background but an infinitesimal percentage of the overall dynamic range. Therefore, we set a threshold for effect size, which was the maximum, background-subtracted YFP observed in a given cell line’s full dataset divided by 30, i.e. ~3% of the cell line’s dynamic range, consistent with our biological intuition that small absolute changes in pathway activation should not drive pairwise response classification. This threshold achieved two objectives, nearly eliminating spurious identification of strong pairwise interactions due to small variations in background and most closely matching a manual classification of pairwise interaction type.

Nonetheless, the correct, automatic classification of some rims was not possible with this approach, perhaps due to a liquid-handling error specific to one or a set of measurements. For these 13 of the 385 total pairs (<4% of the full dataset of 7 cell lines with 45 pairs and 10 controls pairs each), we determined a more reliable estimate of IC value by either setting IC to 0 for the null hypothesis of saturated additivity, selecting a less noisy point on the rim for classification, or calculating IC from independent measurements of that same pair. A full description of these pairs and their manual classification is included below.

##### Rim classification corrections

Confident, automatic classification of all pairwise interactions posed a significant challenge. Measurements were limited to three or four biological replicates while small robotic errors, such as dropped tips or cross-well contamination, added unusually large noise to a small subset of the data. While the analysis pipeline outlined above minimized effects of technical and biological noise by rescaling, and randomization of experiment layout distributed any systematic errors more evenly, the automatic classification of pairwise interactions did not match our intuition in a small number of cases (13 of the 385 rims). Here we describe those cases, why they resisted automatic classification, and how we manually corrected the classification.

First, for two measures of pathway activation to be considered different, the ranges of their respective biological replicates had to be non-overlapping. However, a single outlier among three or four points may cause these ranges to overlap, despite significant differences in the median. Thus, a single erroneous measurement can cause plausible non-additive interactions to be classified as additive. For example, in NMuMG cells, BMP2 and BMP4 combined with GDF5 or GDF7 produced antagonism distinct from GDF6’s lack of effect of BMP2 (Figure S2E). Similarly, in mESC, BMP2 and BMP4 interacted very differently with BMP10 than with other activating ligands, and BMP2 was antagonized by GDF5, but not GDF6 (Figure S3D). However, despite these qualitative differences, all these rims were classified as additive due to a single outlier. For correct classification of these rims, the requirement for non-overlap was removed for the specific points on the rim where the pairwise interaction was clear (cf. asterisks in Figure S2E).

Second, a few rims were classified as strong synergy due to a small increase in pathway activation at subsaturating concentrations. However, synergy manifesting exclusively at extreme ligand ratios or small absolute effect sizes is unlikely for true synergistic interactions. Moreover, given the width of the distribution of median YFP measurements, multiple samples from the same distribution could often, by random chance, produce non-overlapping measurements. For example, in NMuMG cells, despite the similarity of responses to BMP9 paired with GDF5 or GDF6, the latter was classified as synergy due to lack of overlap at low activation levels (Figure S2E). Similarly, in the BMPR2 knockdown cell line, BMP2 paired with BMP5, BMP6, and BMP7 showed synergy at low activation levels, despite not much clear difference from BMP4’s additive interaction with BMP5 (Figure S5G). These small variations were not considered sufficient evidence for a strong claim of synergy, and these pairwise interactions were classified as additive, the null hypothesis for all pairs.

Finally, two unexpected edge cases emerged in the ACVR1 knockdown. The combination of BMP4 with BMP9 generated both positive and negative IC values, though the positive IC values were larger (Figure S4D). However, the negative IC effect was larger in absolute terms and consistent with strong antagonism observed in similar rims, such as BMP5 with BMP9. Therefore, this pair was summarized by only its largest negative IC value. Lastly, BMP2 with BMP10 appeared to be a borderline suppressive interaction. A closer examination of this pair at higher concentrations and more ligand ratios confirmed the suppression between BMP2 and BMP10, and this IC value was used to classify these ligands (Figures S4E,F).

##### Utility of the Interaction Coefficient

The Interaction Coefficient (IC) enabled several parts of the analysis presented here. First, as a dimensionless quantity, it allowed easy comparison between cell lines with drastically different dynamic ranges, either due to differences in sensor behavior or data being collected on different cytometers. Second, as a quantitative, rather than qualitative, metric, IC allowed “softer” classification of pairwise interactions, since small amounts of noise could possibly generate an observation of a weak, but not strong, antagonistic interaction. Third, IC reliably distinguishes distinct behavior types for a wide array of individual ligand doses, since pairwise data are compared to individual data compared at similar doses.

The few difficulties of using the IC do not limit its usefulness in this study. One possible drawback to computing IC as described here is the discontinuity of the metric, introduced to avoid divergence when *f(A)=0* or f(A)f(B). However, these discontinuities preserve better our intuitive classifications of antagonism and suppression. Another challenge is that IC is computed by sampling many ligand ratios, computing IC for each ratio, and then summarizing the pairwise behavior with the largest magnitude IC value, whether positive or negative. However, this is a feature of all synergy metrics, which are defined with respect to the concentrations and ratio at which the pair is studied. Rather, this highlights the importance of reporting synergy alongside the concentration regime that was sampled and recognizing that all estimates of non-additivity represent lower bounds.

Ultimately, IC agrees with preexisting synergy metrics, while providing improvements specific to this application. Defining a synergy metric as distinct regimes normalized to their maximum effect size as implemented by (Yeh et al., 2006), provides the basis for IC, though with a unique definition of additivity. Similarly, IC summarizes the same features described by Relative Ligand Strength and Ligand Interference Coefficient in another study of BMP pairwise interactions (Antebi et al., 2017), but with two small differences. First, an RLS value less than one previously classified an interaction as ratiometric (i.e. antagonistic), but our study showed that not all non-activating ligands (e.g. RLS ~ 0) antagonized activating ligands, as captured by IC’s comparison of the combination response to activation by the stronger ligand alone. Second, nonzero LIC values earn a classification of imbalance or balance, but depend on the assumption that each ligand is individually saturating, such that any increase or decrease relative to that amount is noteworthy. However, as observed in mESCs (Figure 4A), not all ligands appeared to saturate the pathway. The assumptions underlying IC allow its use at subsaturating ligand concentrations, as in the case of BMP9 in mESCs.

#### Determining equivalence groups

To classify the functional similarity of ligands, we sought a quantitative method to summarize differences between each ligand’s pattern of individual and pairwise activation. To this end, we used hierarchical clustering to summarize the hierarchy of possible ligand groupings. First, we computed ligand differences as the Euclidean distance between each ligand’s eleven features in a given cell line (i.e. nine IC values in pairs with the nine other ligands, one IC value in pair with itself, and one RLS value). RLS is multiplied by two for reasons described below. Next, we agglomeratively clustered ligands with complete linkage, such that the differences between two clusters is equal to the distance between their two most dissimilar members. Finally, we cut the resulting dendrogram to generate “monochromatic clusters” (cf. (Segrè et al., 2005)), such that ligands in the same group had similar activation strengths and had the same interaction type (i.e. color of synergistic, additive, antagonistic, suppressive), though not necessarily interaction strength, with all other ligands. The definition of our metrics allowed us to define a distance threshold of 1, which roughly corresponds with the desire for monochromaticity. Specifically, a distance of 1 corresponds with a difference of 1 in IC, which is the distance between the different regimes of IC behavior and therefore guarantees a qualitative difference in pairwise interaction or corresponds to a change of 0.5 in RLS (when RLS has been multiplied by 2), which is the difference between a strong and weak activator. Thus, most groups are defined by clusters at a distance of 1, with some small adjustments to preserve monochromaticity.

##### Global equivalence groups

While the dendrogram of ligand differences across multiple cell types (Figure 6A) provides a complete summary of global ligand differences, we sought to summarize those differences by placing ligands in global equivalence groups. When determining global equivalence groups, each ligand has seven times as many features (i.e. IC and RLS values) than when determining equivalence in a single cell line. To maintain a similar threshold, we increased the squared distance threshold seven-fold from 1 to 7, and cut the tree at a Euclidean distance of 7. The five resulting equivalence groups are in fact more robust to choice of distance than other possible groupings, as they are consistent with the widest possible range of thresholds.

However, this threshold increase allows ligands in the same global equivalence group to have small differences in pairwise interactions, meaning the global equivalence groups are not strictly “monochromatic.” Due to the difficulty of confidently classifying individual response pairs, we decided that a determination of *global* inequivalence could not be based on a single pairwise measurement, so the increased threshold requires multiple observations of difference to put ligands in distinct groups.

The three ligands whose global equivalence would be most affected by lowering the threshold (i.e. making it more sensitive to individual pairwise classifications) are GDF5, GDF6, and GDF7. Identifying pairwise interactions for these three ligands was particularly challenging because they are non-activators. In the analysis of individual cell lines, GDFs were placed in separate equivalence groups only when they differed in their ability to produce antagonistic interactions (NMuMG cells, mESCs, ACVR1 knockdown, ACVRL1 ectopic expression) or when they differed in strength of antagonism (BMPR1B ectopic expression). However, a ligand’s inability to produce an antagonistic interaction may not reflect its intrinsic properties, but instead indicate that it is present at too low a concentration. (As discussed in the definition of the Interaction Coefficient, the IC can vary with ligand concentration and therefore represents only a lower bound.) For non-activating ligands, it is difficult to determine which concentrations are sufficiently high and, in particular, whether all non-activators should be used at the same concentration. Indeed, GDF dose-responses in the only cell context where they activated, BMPR1B ectopic expression, showed significant differences in the three ligands’ EC_50_ values (Figure S6E, inset). Therefore, before confidently placing GDFs in separate global equivalence groups, the lack of antagonism or suppression in some cases should be confirmed by repeating experiments with a large range of GDF concentrations.

#### BMP Mathematical Model

##### Model assumptions and justification

Dimeric BMP ligands activate the BMP pathway by complexing with two Type I and two Type II receptors. Once the complex is assembled, constitutively active Type II receptors phosphorylate and activate Type I receptors, which enzymatically activate SMAD1/5/8 molecules, generating a pool of pSMAD that translocates to the nucleus to promote gene expression. To focus on a minimal number of pathway components and steps, our model of BMP pathway activation considered only one-step formation of a trimeric signaling complex, containing a ligand, a Type I receptor, and a Type II receptor. In the model, each ligand binds each receptor dimer with a unique affinity and activates with a unique activity. Ligand specific-activity is a well-documented phenomenon in BMP signaling, though the mechanism is not clear (Nickel and Mueller, 2019).

This simplified model neglects certain details of BMP pathway activation, which decreases model complexity without significant loss of explanatory power for our *in vitro* system. Specifically, one-step complex formation overlooks the stepwise assembly of BMP signaling complexes. However, summarizing a two-step binding process with a single phenomenological parameter does not limit the possible pairwise interactions allowed by the pathway architecture (Su et al., 2020). The reduced model also neglects the pentameric nature of the full BMP signaling complex, where one BMP dimer binds two Type I and two Type II receptors. This model also does not include the other secreted and membrane-bound molecules that can influence signaling complex formation, such as secreted ligand antagonists (e.g. Twsg1, Noggin, Chordin), co-receptors (e.g. RGMA, ENG), pseudo-receptors (e.g. BAMBI), or other inhibitors (e.g. Fst). However, siRNA targeting the most highly-expressed of these molecules in NMuMG, including Twsg1, Fst, and Rgmb, had no effect on pairwise interactions in NMuMG (Antebi et al., 2017).

The model also assumes that receptor expression is effectively constant (i.e. *A*_*j*_^*0*^ and *B*_*k*_^*0*^, defined below) while pathway activation is produced. While some BMP receptors appear to be continuously endocytosed even in the absence of ligands (Hartung et al., 2006), endocytosed receptors can also continue to signal in endosomes and be recycled back to the cell membrane (Ehrlich, 2016; Gleason et al., 2014), in addition to being degraded in lysosomes and replaced by the synthesis of new receptors. Moreover, blocking endocytosis has also been reported to have little effect on Smad1/5/8 phosphorylation (Ehrlich, 2016). Thus, the various mechanisms which remove BMP receptors from the cell membrane do not necessarily remove them from the pool of actively-signaling receptors, suggesting that endocytosis need not violate the simplifying assumption of near constant receptor expression.

Nonetheless, even if endocytosed receptors can continue to signal, it’s not guaranteed that the other processes of receptor degradation or synthesis will maintain the overall receptor expression level. However, less is known about the timescales of these addition and removal processes, so it remains unclear whether ligand stimulation perturbs steady-state receptor expression and, if so, how much and for how long. Previous work (Figure S3F in (Antebi et al., 2017)) showed that BMPR2 protein levels had no significant changes (i.e. overlapping standard deviations) for up to 12 hours after ligand addition in NMuMG (the main cell line used in this work), suggesting the net effect of these unknown processes could effectively maintain receptor expression at a constant level.

(Antebi et al., 2017) also showed (Figure 3B) sustained accumulation of YFP well past the 24 hour time point used in our experiments. Knowing that YFP fold change at 24 hours matches pSmad levels at 20 minutes, we interpret this sustained accumulation as either the delayed readout of short-term dynamics (in which case, assuming no change in receptor expression is more plausible, given limited time for transcriptional feedback) or sustained, long-term dynamics (in which case, total receptor expression is likely also sustained). However, in the latter case, we recognize that it is possible that compensatory shifts in other pathway components may mask a reduction in total receptor amount.

##### Model equations and solution

To model BMP pathway activation, we used mass-action kinetics to describe the one-step formation of tripartite signaling complexes (*T*_*ijk*_), where one ligand (*L*_*i*_) binds one Type I receptor (*A*_*j*_) and one Type II receptor (*B*_*k*_) with a complex-specific affinity (*K*_*ijk*_). Each complex also has a distinct activity (*ε*_*ijk*_) with which it phosphorylates downstream SMAD proteins, and each complex contributes to the overall measured output (*S*). We assume that binding kinetics reach steady-state more rapidly than complexes activate the pathway. We also assume that ligand concentration, provided by a large reservoir of cell media in experiments, is effectively constant. As shown in (Su et al., 2020), this results in the final equations:

$$S=\sum_{ijk}\epsilon_{ijk}T_{ijk}\;T_{ijk}=K_{ijk}L_{i}^{0}A_{j}B_{k}\;A_{j}=A_{j}^{0}-\sum_{ik}T_{ijk}\;B_{k}=B_{k}^{0}-\sum_{ij}T_{ijk}$$

where $L_{i}^{0}$, $A_{j}^{0}$, and $B_{k}^{0}$ correspond to the starting concentrations of these free species. Thus, when the receptor and ligand environment are specified (i.e. $L_{i}^{0}$, $A_{j}^{0}$, and $B_{k}^{0}$ are known), pathway activation can be solved for, as a function of independent variables are *K*_*ijk*_ and *ε*_*ijk*_. The implicit dependence of *S* on the model parameters can be solved by constrained optimization, implemented by the Python package promisys (Su et al., 2020).

##### Parameter fitting

To better constrain the fit parameters, we pooled datasets that could be fit by the fewest parameters, keeping in mind that the number of parameters scales with the number of ligand and receptor components. To this end, the model was fit to five ligands signaling in cell lines that expressed only five of the seven BMP receptors. Specifically, we focused on BMP4, BMP7, BMP9, BMP10, and GDF5, representing each one of the global equivalence groups and neglecting possible differences between GDFs. We further selected the NMuMG and receptor knockdown datasets, which express the fluorescent BMP reporter from the same genomic locus and express at most five BMP receptors (ACVR1, ACVR2A, ACVR2B, BMPR1A, BMPR2). Receptor expression in these four cell lines was also measured by the same RT-qPCR protocol.

Using the model, our measurements of BMP signaling can be modeled by *S* and its dependence on *L*_*i*_^*0*^, *A*_*j*_^*0*^*, B*_*k*_^*0*^, the known ligand and receptor environments, and *K*_*ijk*_ and *ε*_*ijk*_, the unknown affinities and activities that are fit. However, the data carry various units that also appear in the fit parameters. *S* is measured in units of background-subtracted median YFP, *L*_*i*_^*0*^ in units of ng/mL, and *A*_*j*_^*0*^ and *B*_*k*_^*0*^ in relative expression units from RT-qPCR abundance normalized to a housekeeping gene. The fit parameter units depend on these values in the following way (where *[=]* denotes having the same units):

$$T_{ijk}[=]A_{j}\;B_{ijk}[=]\frac{1}{L_{i}A_{j}}\;\epsilon_{ijk}[=]\frac{S}{A_{j}}$$

The implicit units in the fit parameters influenced the parameter fitting in the following two ways. First, any global transform of *S,* $L_{i}^{0}$, $A_{j}^{0}$, or $B_{k}^{0}$ affects absolute parameter values to reflect the change in units associated with the transformation, but does not affect the overall fit. Therefore, for convenience, all values of *S*, $A_{j}^{0}$, or $B_{k}^{0}$ were normalized to a maximum of 1, dividing by the maximum YFP or relative expression value, respectively, across all four cell lines. Second, the lack of meaningful units for the *K*_*ijk*_ and *ε*_*ijk*_ parameters made it difficult to define biologically-reasonable constraints on the absolute values of these parameters. In principle, the values of *K*_*ijk*_ and *ε*_*ijk*_ may take any nonnegative value, but they cannot physically vary over an arbitrary number of orders of magnitude. Therefore, *K*_*ijk*_ and *ε*_*ijk*_ were bounded within six orders of magnitude. To find reasonable upper and lower bounds, a subset of the data was fit with strictly positive *K*_*ijk*_ and *ε*_*ijk*_ values, which used a method similar to the one described below but did not constrain parameters and produced dozens of equivalently good solutions. These sets of solutions showed most best fit parameter values for *K*_*ijk*_ and *ε*_*ijk*_ ranged between 10^−4^ and 10^2^ when *S* and receptor expression were normalized between 0 and 1.

A final consideration in model fitting was the nature of RT-qPCR measurements. These values introduce significantly larger error than $L_{i}^{0}$, which is directly controlled, and *S*, which is a median across hundreds of cells. At the high C_t_ values of these relatively low abundance receptors, estimates of the same level of expression could vary as much as 2-fold, assuming a 3% difference of efficiency amplified over 25 cycles and do not report final protein levels, which may differ from reported mRNA levels. As relative receptor levels are crucial in the model, a rescaling parameter, bounded by a 3-fold increase or decrease, was added for each receptor, to reflect the uncertainty of the RT-qPCR estimate of protein expression. Thus, the values passed to the model are instead:

$$A_{j}^{0}=\rho_{j}A_{j}^{0,qPCR}\;B_{k}^{0}=\rho_{k}B_{k}^{0,qPCR}$$

where $A_{j}^{0,qPCR}$ and $B_{k}^{0,qPCR}$ are total receptor expression measured by RT-qPCR and *j* and *k* are their respective rescaling parameters. To fit *K*_*ijk*_ and *ε*_*ijk*_, we used SciPy’s least squares function to minimize the error between observed and model-predicted *S*, when $L_{i}^{0}$, $A_{j}^{0}$, and $B_{k}^{0}$ were specified and *K*_*ijk*_*, ε*_*ijk*_, *⍴*_*j*_, and *⍴*_*k*_ freely varied over the ranges described above. Each parameter fitting run was initiated with a distinct, randomly-chosen initial condition and resulted in an equal number of distinct solutions, each with some total error. Filtering out solutions whose error changed the type, rather than strength, of pairwise interactions between ligands, we identified a set of 9 solutions that most closely matched the data’s qualitative behavior (Figure S8). We selected best fit parameter estimates that simultaneously had low error in general as well as low error for specific hard-to-fit pairs (i.e. instances of suppression and synergy in NMuMG and the ACVR1 knockdown cell line). The thresholds for these four criteria (Figure S8A) were selected to include the smallest range around the empirically observed value while still finding some solutions that could meet all four criteria simultaneously. The values for the fit parameters shown in Figure S9 are reported in Table S5.

## Supplementary Material

1

2 Table S1: Recombinant BMP ligands and concentrations

## Figures and Tables

**Table:** Key Resources Table

REAGENT or RESOURCE	SOURCE	IDENTIFIER
**Antibodies**		
**Bacterial and Virus Strains**		
**Biological Samples**		
**Chemicals, Peptides, and Recombinant Proteins**		
Fetal Bovine Serum	VWR	311K18
Fetal Bovine Serum (ES qualified)	ThermoFisher	16141
Leukemia Inhibiting Factor	ThermoFisher	Cat#ESG1107
RNAiMAX	ThermoFisher	Cat#13778075
Lipofectamine LTX	ThermoFisher	Cat#15338100
Trypsin (0.25%)	ThermoFisher	Cat#25200056
Accutase	ThermoFisher	Cat#A1110501
BMP ligands (see Table S1)	R&D	See Table S1
**Critical Commerical Assays**		
RNAeasy mini kit	QIAGEN	Cat#74104
iScript cDNA synthesis kit	BioRad	Cat#1708890
IQ SYBR Green Supermix	BioRad	Cat#1708882
SsoAdvanced Universal probes Supermix	BioRad	Cat#1725281
**Deposited Data**		
All flow cytometry data	This paper	http://dx.doi.org/10.22002/D1.1693
All RT-qPCR data	This paper	http://dx.doi.org/10.22002/D1.1693
RNA-Seq (NMuMG wildtype, mESC E14 strain)	Antebi, et al. 2017	GSE98674
BMP10 protein sequence	UniProt	Q9R229
BMP2 protein sequence	UniProt	P12643
BMP4 protein sequence	UniProt	P21275
BMP5 protein sequence	UniProt	P49003
BMP6 protein sequence	UniProt	P20722
BMP7 protein sequence	UniProt	NP_031583
BMP8 protein sequence	UniProt	P34821
BMP9 protein sequence	UniProt	AAD56961
GDF5 protein sequence	UniProt	P43027
GDF6 protein sequence	UniProt	P43028
GDF7 protein sequence	UniProt	NP_038555
ARCHS RNA-Seq	Lachmann et al. 2018	https://amp.pharm.mssm.edu/archs4
**Experimental Models: Cell Lines**		
NMuMG	ATCC	CRL-1636
NMuMG Sensor Line	This paper	N/A
mESC Sensor Line	Antebi, et al. 2017	N/A
NMuMG Sensor Line overexpressing BMPR1B	This paper	N/A
NMuMG Sensor Line overexpressing BMPR1B	This paper	N/A
NMuMG Sensor Line, stable BMPR2 shRNA	This paper	N/A
NMuMG Sensor Line, stable ACVR1 shRNA	This paper	N/A
NMuMG Sensor Line, stable BMPR1A shRNA	This paper	N/A
**Experimental Models: Organisms/Strains**		
**Oligonucleotides**		
siRNA targeting BMP receptors (see Table)	Antebi, et al.2017	See Table
qPCR primers and probes for BMP receptors (see Table)	Antebi, et al. 2017	See Table
**Recombinant DNA**		
pHK004_PB_MLP_BRE_H2B-C_puro	This paper	N/A
**Software and Algorithms**		
MATLAB	MathWorks	N/A
Python	Python Software Foundation	N/A
Code for analysis and figures	This paper	http://dx.doi.org/10.22002/D1.1693
Easyflow (MATLAB-based flow cytometry analysis software)	Antebi lab	https://doi.org/10.5281/zenodo.5761540
Promisys (Python code for simulating ligand-receptor interactions)	Su et al.	http://dx.doi.org/10.22002/D1.1692
**Other**		
SMARTvector BMPR2-targeting shRNA viral particles (mEF1a-RFP; see Table S2)	Dharmacon	V3SM7598-10EG12168
SMARTvector BMPR1A-targeting shRNA viral particles (mEF1a-RFP; see Table S2)	Dharmacon	V3SM7598-10EG12166
SMARTvector ACVR1-targeting shRNA viral particles (mEF1a-RFP; see Table S2)	Dharmacon	V3SM7598-10EG11477
SMARTvector GAPDH-targeting shRNA viral particles (mEF1a-RFP)	Dharmacon	S10-002000-01
SMARTvector Non-targeting shRNA viral particles (mEF1a-RFP)	Dharmacon	S10-005000-01

## Highlights

- Systematic pairwise analysis of BMP ligands reveals diverse combinatorial effects
- Receptor expression profile (context) controls how ligands signal in combinations
- Contextual equivalence groups classify ligands and explain previous observations
- A model based on alternative ligand-receptor complex formation can explain data
