# Supplementary material for: The context-dependent, combinatorial logic of BMP signaling
Source: Cell Syst. Author manuscript; Available in PMC 2023 May 18. (PMC9127470; doi:10.1016/j.cels.2022.03.002)
Supplement: 1 [file NIHMS1799293-supplement-1.pdf]

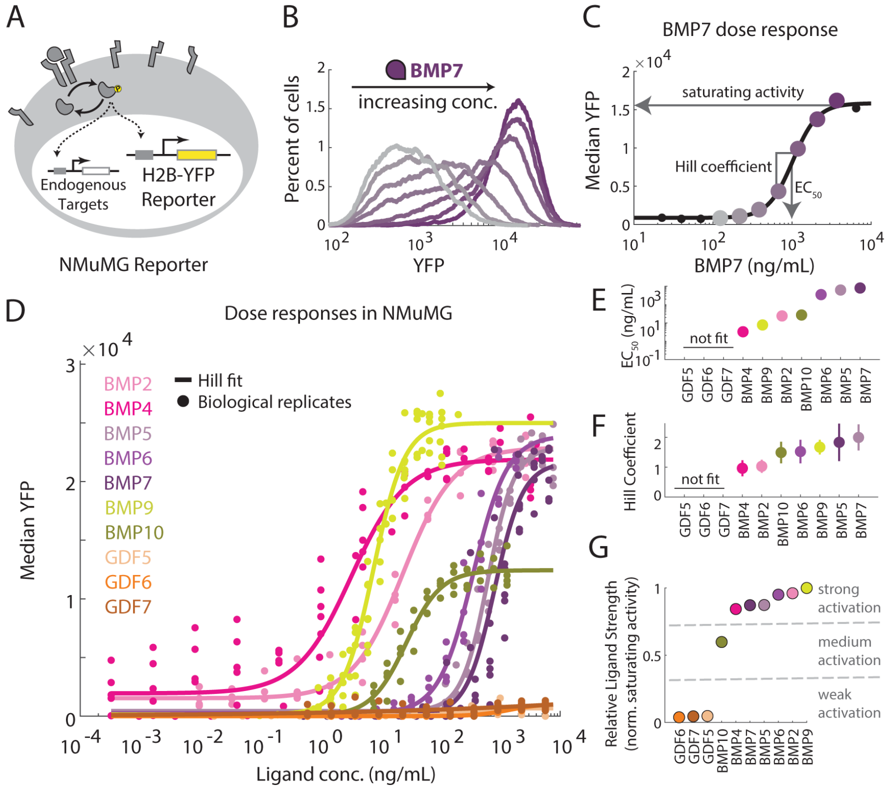

Figure S1: Quantitative dose-responses reveal individual ligand differences.

A. NMuMG reporter cells include a stably-integrated BMP response element driving expression of Histone 2B-mCitrine (H2B-YFP).
B. Flow cytometry data (YFP channel) for a BMP7 dose response in the NMuMG reporter cell line shows concentration-dependent increase in YFP. Colors shaded from gray to purple indicate increasing concentrations of BMP7, plotted in (C).
C. Medians of YFP distributions in (B) show the BMP7 dose response, plotted as a function of BMP7 concentrations, colored by the same scheme as in (B). The black line shows the Hill fit of this single biological replicate. Black dots are responses at concentrations not shown in panel B, but acquired in the same manner.
D. Dose responses for ten BMP ligands in the reporter line vary in slope, EC_{50}, and saturating activity. Dots represent three biological repeats, and lines indicate the best fits to Hill functions.
E. EC_{50} values from best fit Hill functions for all activating ligands vary continuously over two orders of magnitude. Parameters for GDF5, GDF6, GDF7, whose weak responses do not resemble Hill fits, were omitted.
F. Hill coefficients for activating ligands range between 1 and 2.
G. Relative Ligand Strength (RLS, saturating ligand activity normalized to the strongest ligand's saturating activity) values show weak, medium, and strong activation levels.

See also Figure 3.

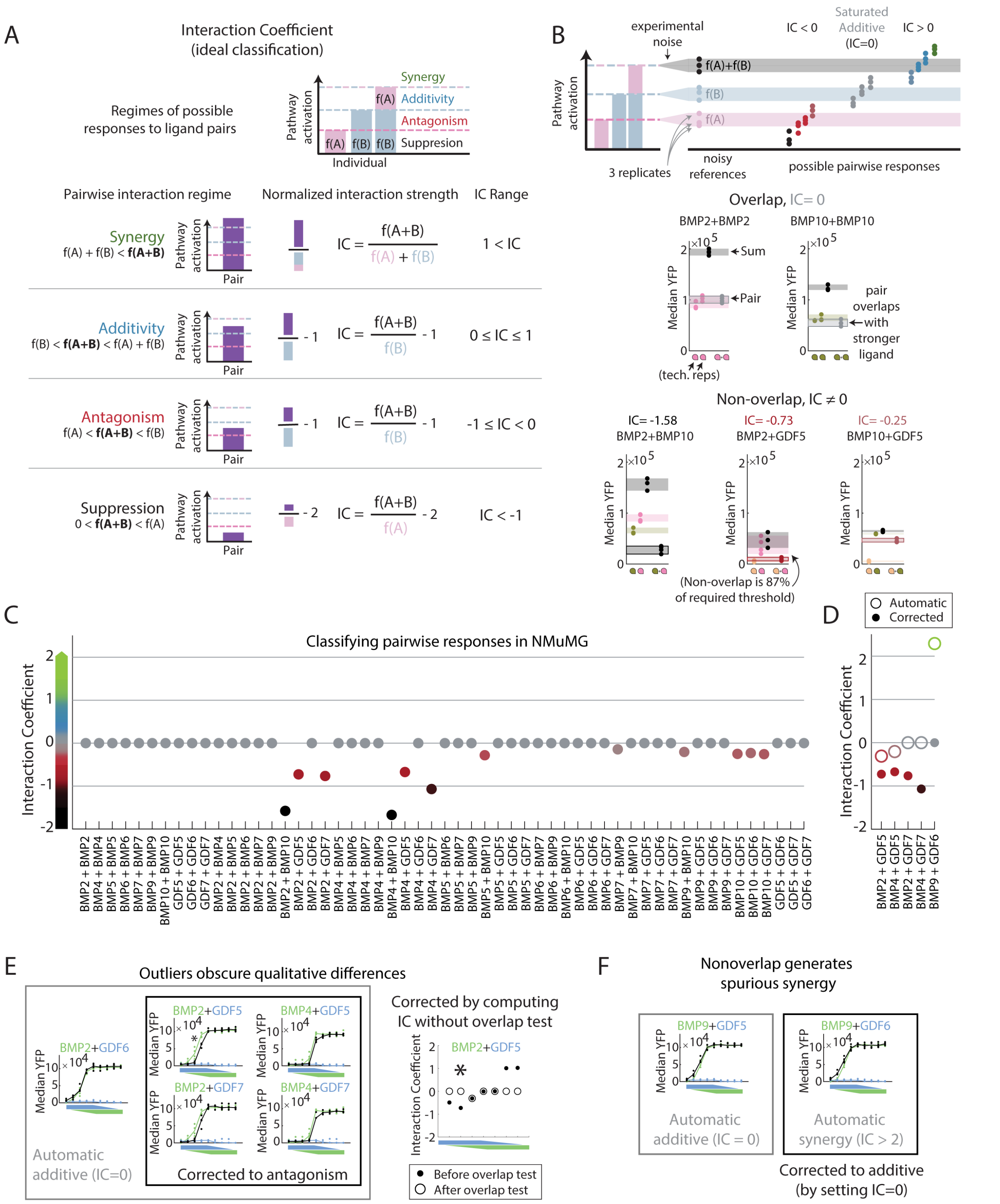

Figure S2: Interaction Coefficient (IC) classifies pairwise interactions relative to individual and summed responses.

A. The Interaction Coefficient (IC) is computed by a discontinuous, piecewise formula corresponding to four regimes of possible pairwise response. These four regimes (synergy,

additivity, antagonism, suppression) are defined by the magnitude of pairwise response relative to the individual responses (i.e. *f(A+B)* relative to *f(A)* and *f(B)*, or their sum *f(A)+f(B)*).

B. While IC quantifies the pairwise effect size, requiring nonoverlap between two groups of measurements filters for more significant pairwise effects. Limited replicates of the stronger and weaker individual ligand responses make it difficult to classify an equally limited sample of the pairwise response as similar or distinct. In our analysis, overlapping ranges of pairwise and individual responses are considered equivalent, such that pairwise responses that overlap with the stronger individual ligand response are classified as saturated additive (IC=0), such as when BMP2 or BMP10 is paired with itself, shown by how the gray transparent rectangle showing the range of pairwise responses overlaps with the pink or green rectangles showing the range of individual responses. Pairwise responses that do not overlap with the stronger individual response can have nonzero IC values, computed on the median of the replicates, as for BMP2 paired with BMP10 and BMP10 paired with GDF5. The response to BMP2 paired with GDF5 has only 87% of the required non-overlap with the individual BMP2 response, due to the larger variance in the BMP2 response, but is manually corrected to an antagonistic interaction (see (E) and STAR Methods).

C. For each ligand pair, one IC value is determined (STAR Methods). Most responses are saturated additive (gray), with limited instances of antagonism (red) with GDF5 and GDF7 and suppression (black) with BMP10.

D. Some rims required a different calculation of IC. The automated (open circle) and corrected (closed circle) values are shown for the five pairs where these changes were made. See (E) and (F) for examples, and STAR Methods for full discussion.

E. To be classified as antagonistic, the full set of biological repeats for a rim response must not overlap with the distribution of measurements for the stronger individual ligand. However, for four of the corrected rims, single outliers created overlap and obscured clear differences between the rim and gradient. For example, BMP2 combined with GDF6 clearly overlaps with the response to BMP2 alone and is saturated additive, while BMP2 and BMP4 combined with GDF5 and GDF7 appear qualitatively different. These pairwise responses (boxed in black) show individual ligand responses (blue and green) that minimally overlap with pairwise responses (black). In each plot, the x-axis corresponds to multiple ratios of the two ligands, as in Figure 2A. To correctly classify these rims, the requirement for nonoverlap was neglected. As an example, the IC value before the overlap test (computed on the median of replicates) and after (where overlapping pairiwe and individual responses are automatically classified as IC=0) are plotted on the right. The ratio with the strongest antagonism (marked by an asterisk) can be recovered by ignoring the nonoverlap requirement.

F. Similarly, nonoverlap between the spread of points for rim and gradient measurements spuriously classifies rims as synergistic, even though very similar rims show significant overlap. BMP9 combines with GDF5 and GDF6 in qualitatively similar ways, despite small differences in whether every point on the rim overlaps with the BMP9 gradient. Thus, BMP9 with GDF6 was classified as saturated additive, to match BMP9 with GDF5.

See also Figure 3.

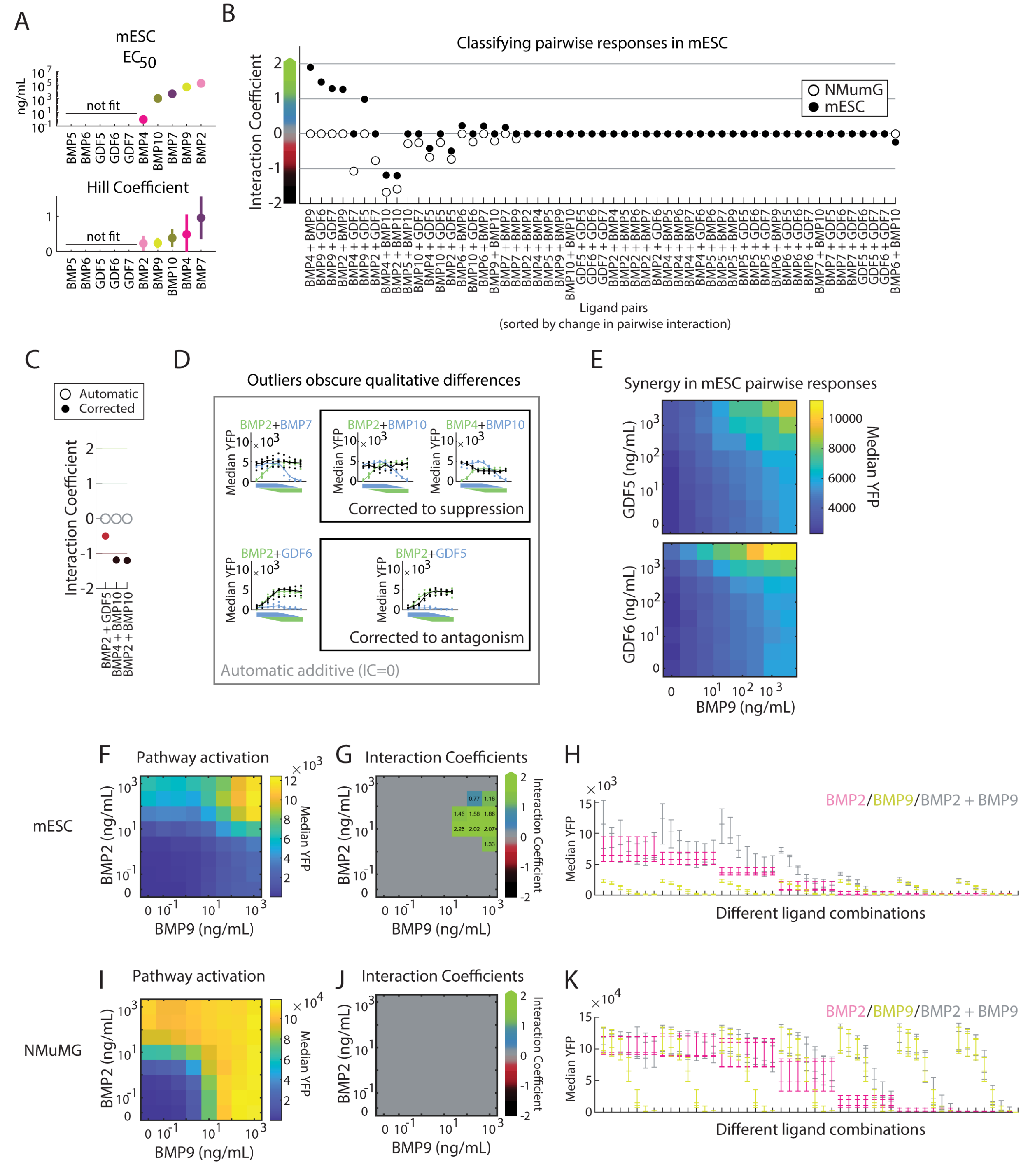

Figure S3: Full quantification of BMP signaling in mESC reveals unique ligand properties.

A. Dose responses to individual BMPs in mESCs were fit to Hill functions to determine best fit parameters and compare ligand behavior. Ligands that do not activate (GDF5, GDF6, GDF7) or do not saturate (BMP5, BMP6) were not fit. Relative to NMuMG cells, Hill coefficients are much lower for mESCs, and EC_{50} values are much higher (cf. Figures S1E,F).

B. Between NMuMG cells and mESCs, a small percentage of pairwise interactions changed a large amount. Most changes represented an increase in IC. IC values for all ligand pairs

activating mESC (closed circle) and NMuMG cells (open circle, as in S2B) are sorted by decreasing change in IC values.

C. Automatic classification of pairwise interactions failed for three rims, where antagonistic and suppressive interactions were misclassified as additive. See Figure S2E for similar examples from NMuMG, and STAR Methods for full discussion.
D. As in Figure S2E, single outliers created overlap between rims and gradients. The corrected IC was computed by ignoring the overlap test for these rims. To show differences between correctly and incorrectly classified pairs, additive interactions of BMP2 with BMP7 or GDF6 are contrasted to BMP2 with BMP10 and GDF5, as well as BMP4 with BMP10.
E. High-resolution matrices of BMP9 with GDF5 and with GDF6 in mESC confirm synergy of BMP9 with non-activating ligands.
F. High-resolution matrix of BMP2 with BMP9 (median of three replicates) in mESC shows a synergistic response, as expected from the synergy of the rim. Heatmap color shows median YFP.
G. Interaction Coefficient (IC) values for each point in the BMP2 plus BMP9 matrix show additive (blue) and synergistic (green) interactions. Nonzero IC values are shown on ligand ratios where they occur.
H. All biological replicates for each ligand combination are plotted alongside the individual ligand responses for the same amount of either BMP2 or BMP9 used in the combination. Synergy occurs at points where the combinatorial responses exceed the sum of the individual responses by more than the variability between replicates.
I. NMuMG responses to the same concentrations of BMP2 and BMP9 as in (F) produce a saturated additive response. Heatmap colored as in (F).
J. No synergy is detected at any combination of BMP2 and BMP9 in NMuMG.
K. Replicates of pairwise responses overlap with replicates of the strongest individual ligand response for all combinations, producing a saturated additive classification for all points in the BMP2 plus BMP9 matrix in NMuMG.

See also Figure 4.

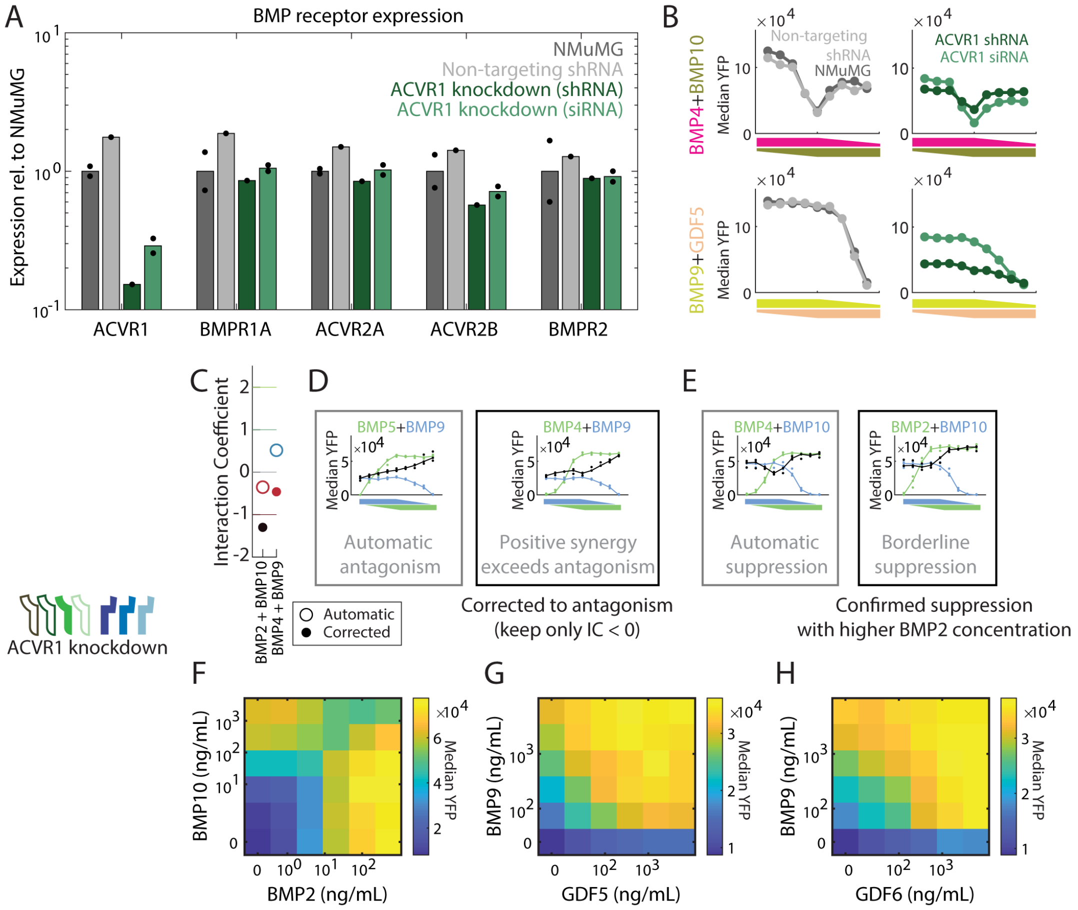

Figure S4: Knockdown of ACVR1 by shRNA phenocopies siRNA knockdown and alters pairwise interactions.

A. RT-qPCR of BMP receptor expression shows potency and specificity of shRNA knockdown of ACVR1 in parental NMuMG reporter line. shRNA knocks down ACVR1 mRNA as potently and specifically as siRNA, while non-targeting shRNA appears to affect all receptors equally. Expression values are normalized to housekeeping gene expression, and then to the average expression of the same gene in NMuMG cells. Dots are biological replicates, and the bar height is either one replicate or geometric mean of two replicates.
B. Responses to ligand pairs sampled at multiple ratios and overall high concentration for the same cell types as in (A) show that non-targeting shRNA has no effect on pairwise responses, whereas knocking down ACVR1 by shRNA produces similar effects as siRNA knockdown, with lower activation by BMP4 and BMP9.
C. Automatic classification of pairwise interactions failed for two rims, as shown in (D) and (E). See STAR Methods for full discussion.
D. BMP4 combined with BMP9 produced a response with synergistic and antagonistic qualities. However, the synergy is a small effect relative to the antagonism, and the rim more closely resembles another strong antagonistic interaction, BMP5 with BMP9. The corrected classification therefore keeps the IC values corresponding to antagonism.
E. BMP2 with BMP10 appeared nearly suppressive, mirroring BMP4 with BMP10. Measuring pairwise interactions at a higher BMP2 concentration (F) revealed a strong suppressive interaction.

Similarly, sampling responses to BMP9 with GDF5 (G) or with GDF6 (H) confirmed the synergistic interactions between these ligands. Heatmap colors show pathway activation as reported by YFP median.

See also Figure 5.

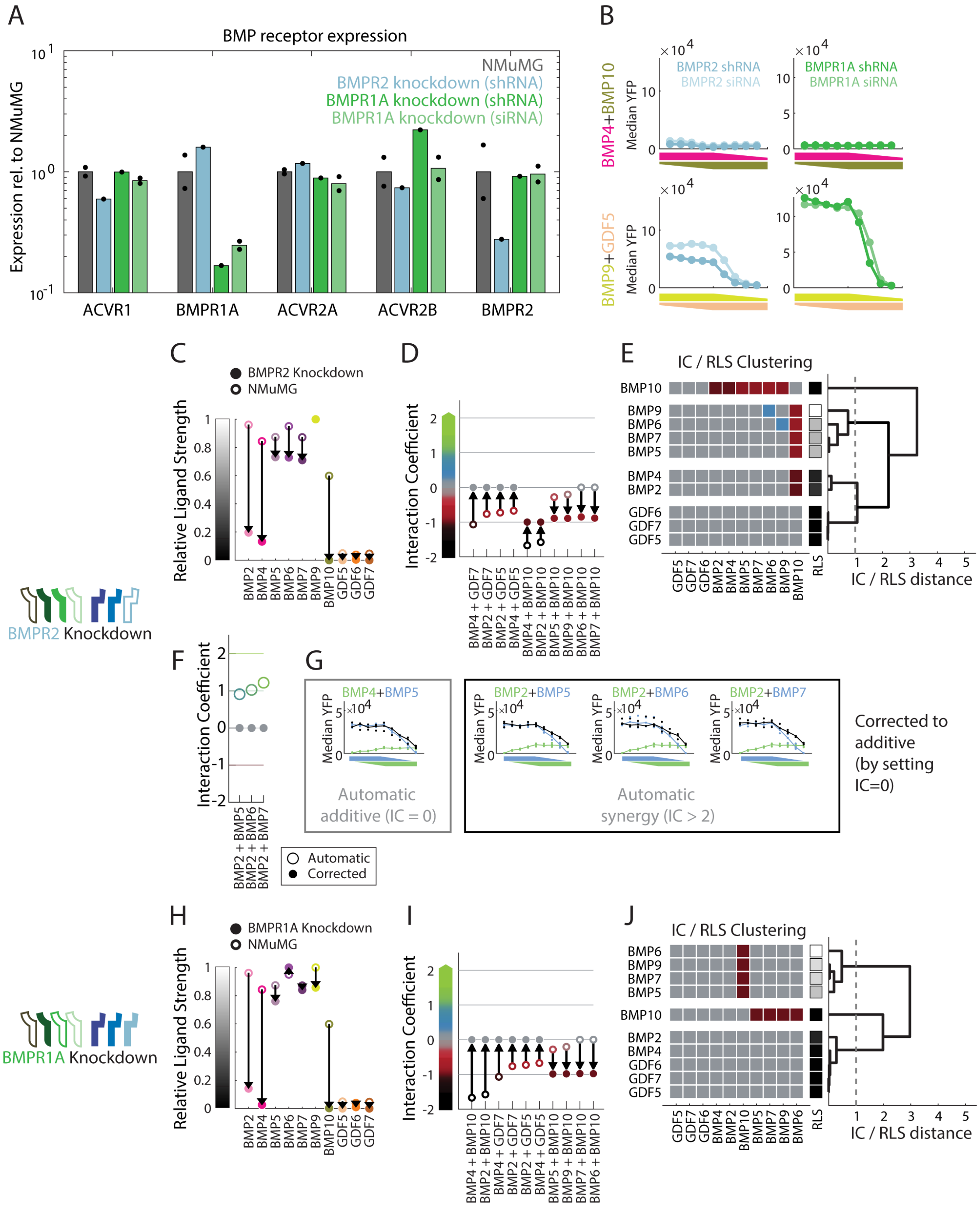

Figure S5: Constitutive shRNA expression knocks down BMPR2 and BMPR1A to produce receptor-specific effects distinct from ACVR1 knockdown.

A. RT-qPCR of BMP receptor expression shows that constitutively expressed shRNA knocks down BMPR2 and BMPR1A potently and relatively specifically, though with small possible increases in BMPR1A and ACVR2B, respectively. Expression values are normalized to housekeeping gene expression, and then to the average expression of the same gene in NMuMG cells. Dots are biological replicates, and bar height is either one replicate or geometric mean of two replicates.

B. Despite apparent perturbations to off-target BMP receptors, shRNA knockdown produces qualitatively similar responses to ligand pairs as the more specific siRNA knockdown. Pairwise responses are sampled at multiple ratios and overall high concentration in NMuMG reporter with BMPR2 and BMPR1A targeted by siRNA or shRNA.

Relative Ligand Strength (RLS) and Interaction Coefficient (IC) values change in specific ways following BMPR2 knockdown (closed circle), compared to NMuMG cells (open circle). (C) BMPR2 knockdown substantially reduces activation by BMP2, BMP4, and BMP10. (D) Antagonism of BMP2 and BMP4 with GDFs is lost, while all BMP10 interactions become strong antagonism. Changes of IC less than 0.5 are not shown. (E) Hierarchical clustering of all IC and RLS values reveals equivalence groups following BMPR2 knockdown. (F) Automatic classification of pairwise responses failed for three ligand pairs in the BMPR2 knockdown dataset, as shown by the automatic (open circle) and corrected (closed circle) measurements. (G) Small nonoverlap between the rim and the gradient is classified as a synergistic interaction, analogous to Figure S2F. The IC values for BMP2 combined BMP5, BMP6, and BMP7 are corrected to 0, to match the qualitatively similar pair, BMP4 with BMP5 (STAR Methods).

RLS and IC also change following BMPR1A knockdown (closed circle) relative to NMuMG cells (open circle). (H) Activation by BMP2 decreases while activation by BMP4 or BMP10 is lost. (I) All interactions of BMP2 and BMP4 with other non-activating ligands become additive, while BMP10 strongly antagonizes all activating ligands. Changes of IC less than 0.5 are not shown. (J) Hierarchical clustering of all IC and RLS values reveals equivalence groups following BMPR1A knockdown.

See also Figure 6.

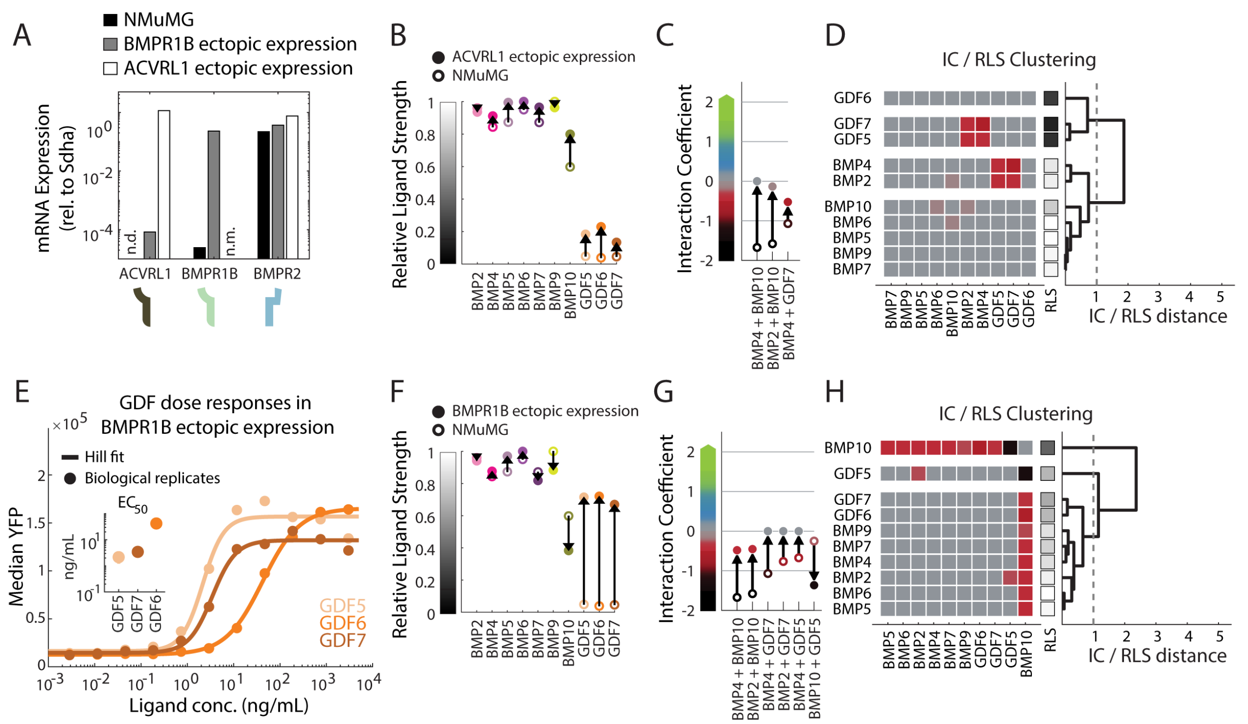

Figure S6: Ectopic expression of missing BMP receptors increases frequency of additive interactions and simplifies ligand equivalence.

A. ACVRL1 and BMPR1B mRNA are not detected (n.d.) or are orders of magnitude lower than housekeeping genes in the original NMuMG reporter. Ectopic expression of ACVRL1 and BMPR1B in the NMuMG reporter increases mRNA levels, quantified by RT-qCPR, to match already expressed BMP receptors, such as BMPR2. BMPR1B levels were not measured (n.m.) in cells ectopically expressing ACVRL1.

B. Ectopic ACVRL1 expression (closed circle) in NMuMG cells (open circle) produces small changes in Relative Ligand Strength (RLS), increasing activation by BMP10 and making GDFs weak activators.

C. Between ectopic ACVRL1 expression (closed circle) and the parental NMuMG reporter (open circle), only three ligand pairs show large changes (|ΔIC|>0.5) in Interaction Coefficient (IC). BMP10's suppression is lost, while GDF7 more weakly antagonizes BMP4. These pairs all include ligands whose RLS value (B) changed a large amount between the two cell lines.

D. Hierarchical clustering of all IC and RLS values reveals equivalence groups following ectopic ACVRL1 expression.

E. GDF5, GDF6, and GDF7 activate NMuMG cells following ectopic expression of BMPR1B, though with different EC_{50} values (inset, Hill fit parameters) that are not apparent in other cell lines where GDFs do not activate.

F. Ectopic BMPR1B expression (closed circle) in NMuMG cells (open circle) produces large changes in RLS, converting GDFs into strong activators and slightly decreasing BMP10's strength.

G. Between ectopic BMPR1B expression (closed circle) and the parental NMuMG reporter (open circle), six ligand pairs show large changes in pairwise interaction strength (|ΔIC|>0.5). These pairs involve ligands whose RLS increased or decreased a large amount. BMP10's suppression becomes antagonism, but GDF5, rather than antagonizing BMP10, suppresses it. Otherwise, antagonism by GDFs becomes mostly additive.

H. Hierarchical clustering of all IC and RLS values reveals equivalence groups following ectopic BMPR1B expression.

See also Figure 6.

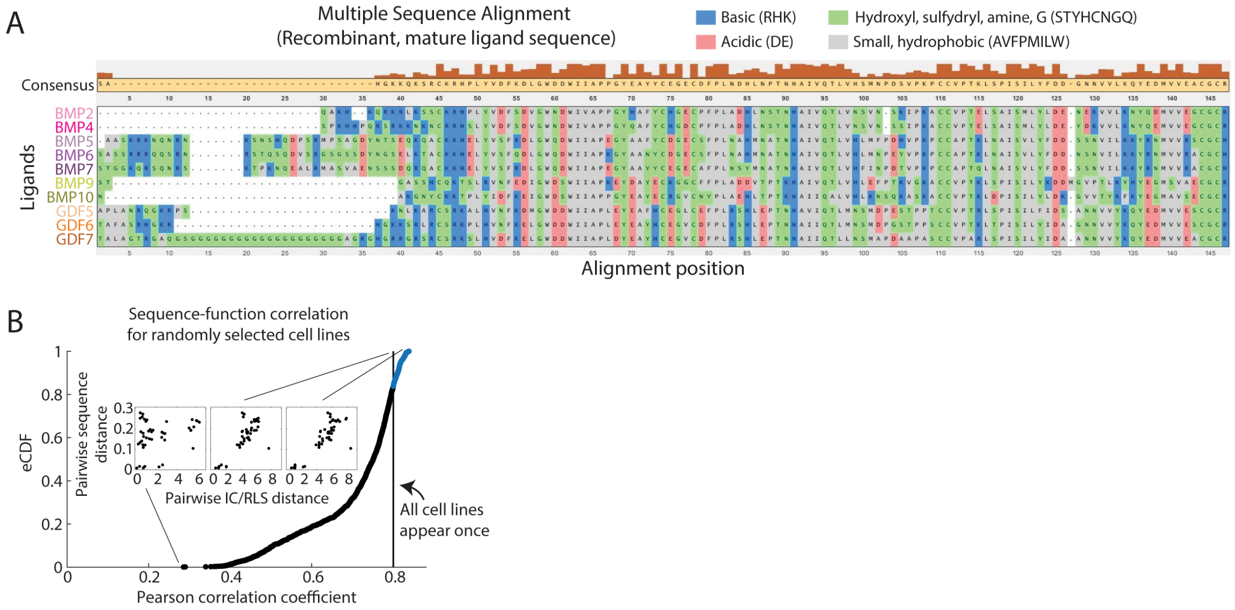

Figure S7: BMP ligand sequence differences correlate with global functional differences.

A. Alignment of recombinant BMP sequences reveals amino acid variations between BMP ligands.

B. To explore whether the better correlation of sequence difference with functional difference (i.e. pairwise IC/RLS distance) observed for all cell lines (cf. Figure 6D) was merely a result of including more datapoints, the correlation coefficient was computed for 10,000 random cell line combinations, i.e. groups of seven cell lines sampled with replacement from the seven cell line datasets. Less than 25% of these combinations produced higher correlation coefficients than including each cell line once and with only a modest increase in correlation coefficient, suggesting that pooling all cell lines improved the correlation not by merely providing more data points but because the multiple cell lines better capture the range of ligand behaviors encoded in the diverse BMP sequences. Scatter plots for the best and worst correlations, as well as the correlation for each cell line appearing once, are shown in the inset.

See also Figure 6.

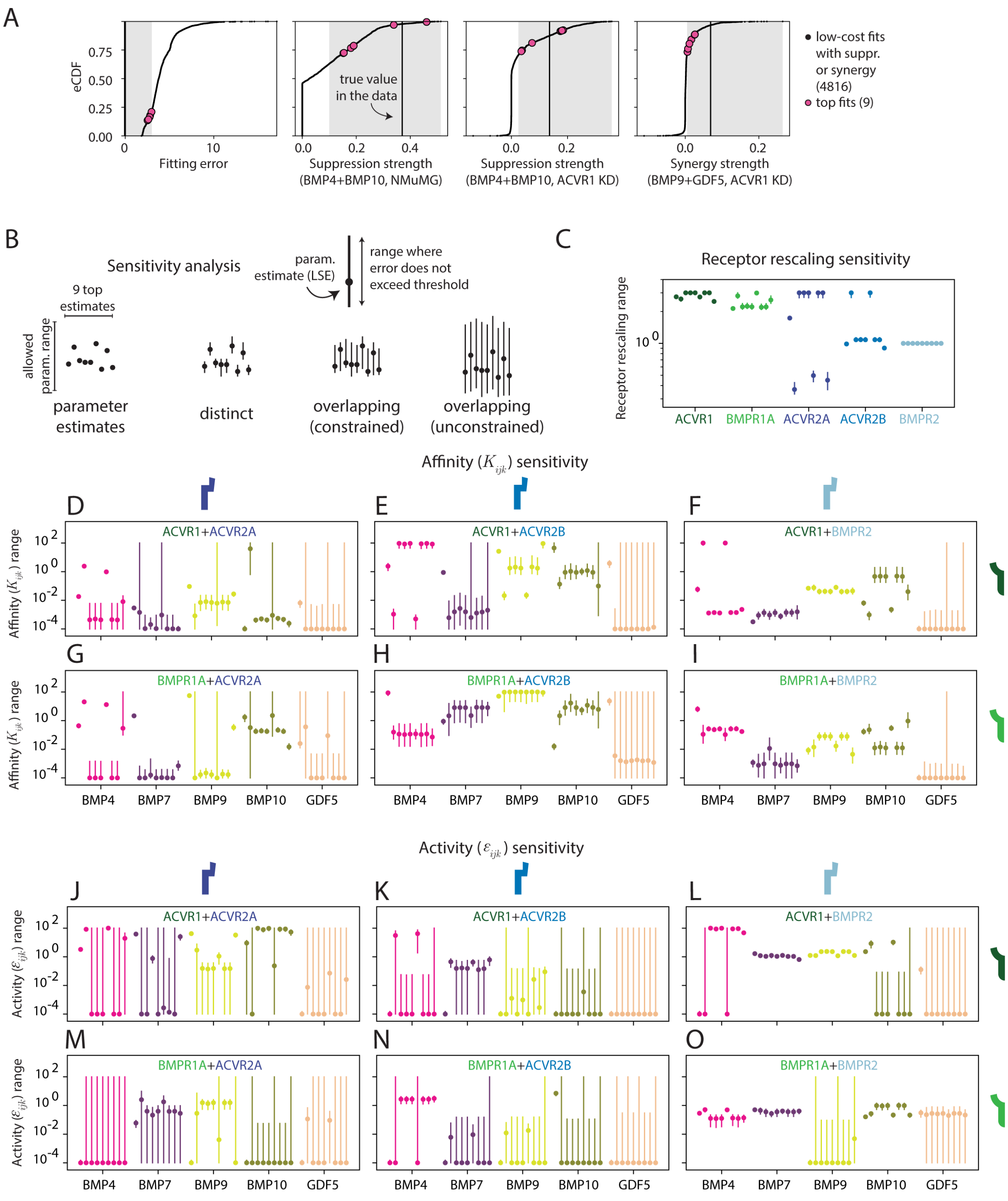

Figure S8: Top parameter fits produce overlapping parameter estimates.

A. Of the 6,000 least-squared error estimates of parameter values generated, 4,816 performed well on at least one goodness-of-fit metric, having overall fitting error less than 2.5 (cutoff for qualitative similarity with the data) or a minimal instance (i.e. IC≠0) of one of the hard-to-fit suppression or synergy interactions (i.e. BMP4+BMP10 in NMuMG, BMP4+BMP10 in ACVR1 knockdown, or BMP9+GDF5 in ACVR1 knockdown). eCDFs (black dots) for these fit metrics across the 4,816 fits show that, despite performing well on individual metrics, most were far

away from the empirically observed value (black line) in general. To find fits that performed reasonably well on all goodness-of-fit metrics simultaneously (pink dots), we selected a cutoff for each metric that included the observed value but somewhat stringently excluded the range of values produced by most fits, ensuring a low fitting error overall as well as the lowest possible error for specific hard-to-fit pairs.

B. For each of the top fits, each parameter value (estimated by minimizing least-squared error, LSE) is plotted alongside the range of values over which that parameter can individually vary without increasing overall goodness-of-fit above the threshold for selecting top fits (as shown with gray region in leftmost plot in (A)). The size of these ranges could span a small region or the entire allowed range of parameter values, perhaps indicating the data do or do not constrain that parameter. Additionally, the overlap or lack thereof across parameter fits indicates how many distinct values of that parameter could fit the data.
C. Goodness-of-fit was highly sensitive to receptor rescaling parameters, which approximate receptor protein expression from qPCR measurements of receptor mRNA. BMPR2 rescaling is fixed at 1, but changes in rescaling for other receptors considerably affects the fits.

Goodness-of-fit was slightly less sensitive to affinity (K_{ijk}) values (D-I) than receptor rescaling parameters. The affinities are shown for each ligand (indicated by the color) binding each receptor dimer (different subplots). Top and bottom rows show ACVR1- and BMPR1A-containing complexes respectively, while columns left to right are ACVR2A-, ACVR2B-, and BMPR2-containing complexes. Affinities for BMPR2-containing complexes (F,I), as well as BMPR1A with ACVR2B (H), are well constrained with good agreement across top parameter fits, with the exception of GDF5 (orange).

By contrast, activity (ε_{ijk}) values are poorly constrained by the data (J-O) and at best could be binned into groups of either high or low activity overall. The only exceptions are the activities of BMPR2-containing complexes (L,O).

See also Figure 7.

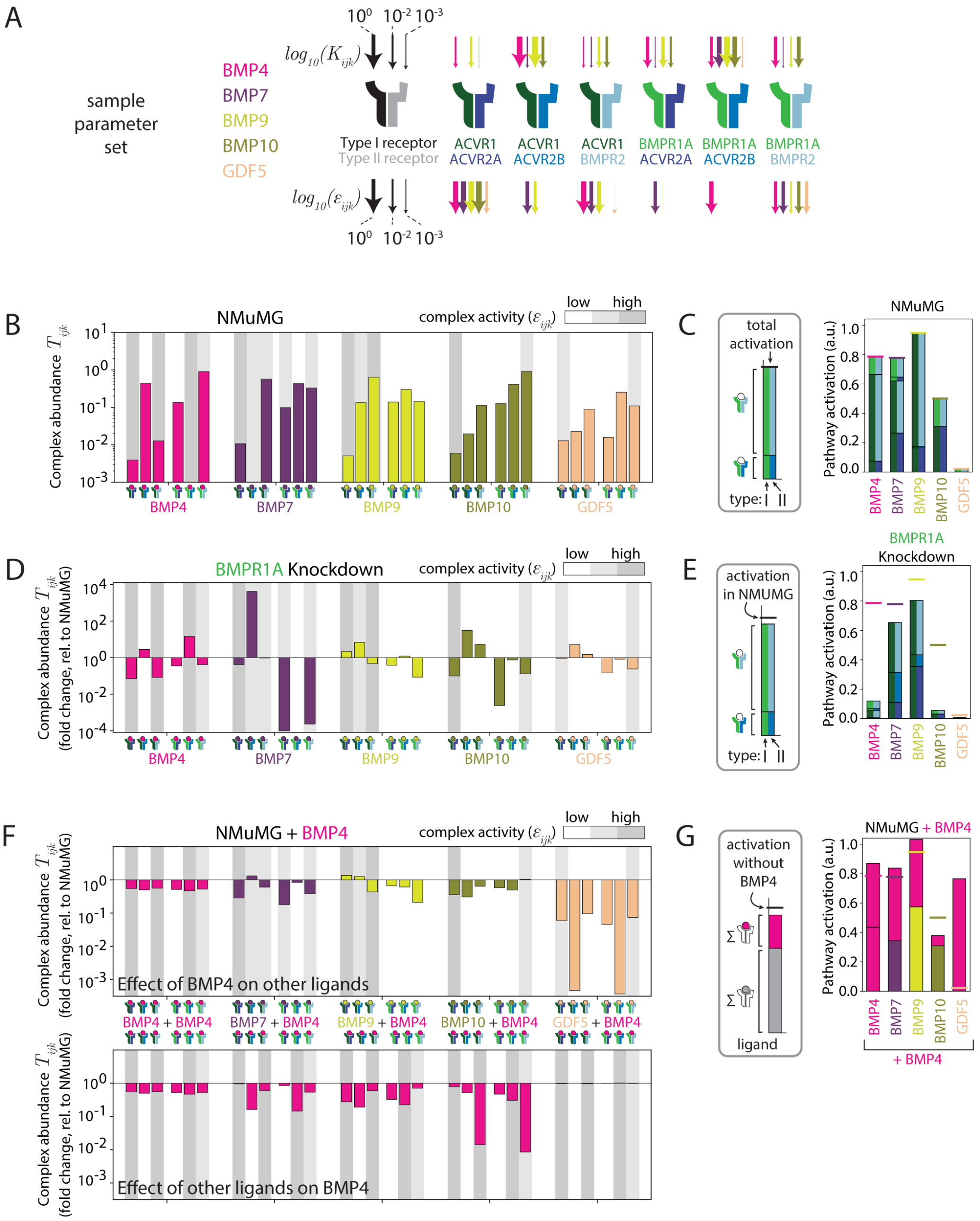

Figure S9: Sample top parameter set shows possible mechanisms for the effects of receptor and ligand context.

A. A sample parameter set that fits all responses to five ligands in four cell lines is represented with line thicknesses. Affinity (above) and activity (below) parameters are grouped by receptor dimer and colored by the ligand species. Ligands bind most receptor dimers but activate few. In many cases, affinity and activity are inversely related, as indicated by thick arrows above and thin arrows below, or vice versa (cf. Figure 7C). The precise values for this parameter set are reported in Table S5.

B. Theoretical abundance of each signaling complex (T_{ijk}) in the mathematical model varies slightly between individual ligands activating NMuMG cells. The possible complexes are

grouped into sets of six, colored by the bound ligand and with the six bars corresponding to the six possible receptor dimers, in the same order as in (A) and shown with the same colors on the x-axis. Grayscale background for each bar indicates the binned (high > 10^0, low < 10^{-2}, medium in between; arbitrary ε_{ijk} units) activity of that complex. Ligands form similar amounts of most complexes (e.g. more BMPR1A-containing complexes than the other Type I receptor), but differ in their ability to activate them.

C. Strongly activating ligands achieve similar levels of pathway activation by activating different amounts of similar receptor dimers, whereas weaker ligands activate fewer receptor dimers. Stacked bars show the theoretical contribution of various signaling complexes. Left and right halves of each unique bar in the stack are colored to indicate unique pairings of the Type I and Type II receptors, respectively.

D. Bars indicate the theoretical fold change, relative to NMuMG cells, in signaling complex abundance following BMPR1A knockdown. Ordering of bars and grayscale background are as in (B). BMPR1A knockdown indirectly increases abundance of ACVR2B-containing complexes, which only BMP7 and BMP9 activate strongly.

E. As in (C), individual ligands activating the BMPR1A knockdown cell line at saturating concentrations utilize different receptor complexes. Unexpectedly, BMP4 and BMP10 produce lower activity overall, despite not signaling strongly through BMPR1A. The loss of BMPR1A leaves excess Type II receptors that shift the binding partners of ACVR1. These rearrangements shift BMP4 and BMP10 to mostly inactive complexes, while BMP7 and BMP9 signal well through all ACVR1-containing complexes.

F. The presence of saturating BMP4 introduces additional BMP4-containing complexes (bottom panel) and alters the abundance of complexes bound to other ligands (upper panel) as receptors are shared between the two ligands. Bars indicate the theoretical fold change, relative to ligands signaling alone, in the amount of each receptor complex. For each ligand pair, the full set of complexes are split between the upper plot (the first ligand of the pair) and lower plot (BMP4, the second ligand of the pair). Ordering of bars and grayscale background are as in (B) and (D). BMP7 and BMP9 lose some activating complexes to BMP4 while also forming new ones, due to their ability to promiscuously activate all ACVR1-containing complexes.

G. Bar height indicates the theoretical contribution of each ligand to overall activation by the ligand pair. Consistent with the effects of competition, overall pathway activation is reduced when BMP4 is combined with BMP10, increased when combined with GDF5, but stays the same, due to sharing of active complexes, when combined with BMP7 and BMP9.

See also Figure 7.

Table S2: shRNA sequences

This table contains the catalog numbers for the shRNA viral particles ordered from Dharmacon (Horizon Discovery) as well as the target sequence for each shRNA.

Target	Catalog #	Source Clone ID	Vector	Gene target sequence
BMPR2	V3SM7592-231979356	V3SVMM10_11917013	pSMART_mEF1a/TurboRFP	ACAAGCAAATACTCCATGC
BMPR2	V3SM7592-234523888	V3SVMM10_14461544	pSMART_mEF1a/TurboRFP	TATCGACCCCGTCCAATCA
BMPR2	V3SM7592-235657177	V3SVMM10_15594830	pSMART_mEF1a/TurboRFP	AGACCATAACACGTGCTCC
BMPR1A	V3SM7592-232280382	V3SVMM10_12248039	pSMART_mEF1a/TurboRFP	GTGTGAAACGCTTGCGGCC
BMPR1A	V3SM7592-233338234	V3SVMM10_13275887	pSMART_mEF1a/TurboRFP	GGAGTGGATCTGGATTGCC
BMPR1A	V3SM7592-235771879	V3SVMM10_15709538	pSMART_mEF1a/TurboRFP	AAATGGAAGTTGCTGTATT
ACVR1	V3SM7592-231384102	V3SVMM10_11321759	pSMART_mEF1a/TurboRFP	ATAAGAGGGTCGATATTTG
ACVR1	V3SM7592-232622298	V3SVMM10_12559952	pSMART_mEF1a/TurboRFP	AATAGGAATTCAATCTGGC
ACVR1	V3SM7592-236015386	V3SVMM10_15953045	pSMART_mEF1a/TurboRFP	GATCACTCGTGTACATCAG

Table S3: siRNA ID numbers

This table contains the siRNA ID numbers for siRNAs (Lifetech) used in this study.

RNA	Source	ID #
BMPR1A siRNA	Lifetech	S201096
BMPR1A siRNA	Lifetech	S201097
BMPR2 siRNA	Lifetech	S63047
BMPR2 siRNA	Lifetech	S63048
ACVR1 siRNA	Lifetech	S61924
ACVR1 siRNA	Lifetech	S61925

Table S4: qPCR primers and probes

This table contains the sequences for the probes, forward primers, and reverse primers used for qPCR of BMP receptors and Sdha (housekeeping gene).

Gene	Forward Primer	Reverse Primer	Probe	Supermix
Bmpr1a	GAGTGGATCTGGATTGCCTTTA	CGCCATTTACCCATCCATACT	/56-FAM/ATTCAGATG/ZEN/GTTCGGCAGGTTGGT/3IABkFQ/	SsoAdvanced Universal probes
Bmpr1b	GCTTGGCTGTCAAGTTCATTAG	CAGCACTTCTGGAGGCATATAG	/56-FAM/ATGAGGTTG/ZEN/ACATCCCACCCAACA/3IABkFQ/	SsoAdvanced Universal probes
Bmpr2	GCAATCTCCCACCGAGATTTA	ACCAGCCGATTTCCAGTTAG	/56-FAM/AGAATGACG/ZEN/GCGCGTGTGTTATCA/3IABkFQ/	SsoAdvanced Universal probes
Acvr1	CACCTGGAAGTTGGCCTTAT	GCTCTTGATTGCGTCTCTTAAAC	/56-FAM/AGCTTGCAT/ZEN/CCTTGGAGTTGCTCT/3IABkFQ/	SsoAdvanced Universal probes
Alk1	TCTGCTTAGACACGACAACATC	GTTCATGGTAGTGGGTGATGAG	/56-FAM/TTCATCGCC/ZEN/TCCGACATGACTTCG/3IABkFQ/	SsoAdvanced Universal probes
Acvr2a	CGTTCGCCGTCTTTCTTATCT	GTCTGGTTGGTTCTGTCTCTTT	/56-FAM/TGCTCTTCA/ZEN/GGTGCTATACTTGGCAG/3IABkFQ/	SsoAdvanced Universal probes
Acvr2b	ATGAGTACATGCTGCCCTTC	CTTAATCGTGGGCCTCATCTT	/56-FAM/AGCTTCAGG/ZEN/AGGTGGTTGTCCAC/3IABkFQ/	SsoAdvanced Universal probes
Sdha	GGATTGCTTCTGTTTGCTTGG	AGTGGGCTGTCTTCCTTAAC	/56-FAM/TGGGCATGT/ZEN/CTCTGAGGGATTGG/3IABkFQ/	SsoAdvanced Universal probes

Table S5: Sample parameter set

This table contains the parameter values for the sample parameter set plotted in Figure S9.

	Ligand	Type I	Type II	Parameter
**Affinity (*K_{ijk}*)**	BMP4	ACVR1	ACVR2A	8.04E-03
	BMP4	ACVR1	ACVR2B	8.45E+01
	BMP4	ACVR1	BMPR2	2.29E-03
	BMP4	BMPR1A	ACVR2A	2.91E-01
	BMP4	BMPR1A	ACVR2B	7.17E-02
	BMP4	BMPR1A	BMPR2	1.73E-01
	BMP7	ACVR1	ACVR2A	1.00E-04
	BMP7	ACVR1	ACVR2B	2.03E-03
	BMP7	ACVR1	BMPR2	1.57E-03
	BMP7	BMPR1A	ACVR2A	6.98E-04
	BMP7	BMPR1A	ACVR2B	8.14E+00
	BMP7	BMPR1A	BMPR2	6.99E-04
	BMP9	ACVR1	ACVR2A	2.77E-02
	BMP9	ACVR1	ACVR2B	9.23E+01
	BMP9	ACVR1	BMPR2	4.49E-02
	BMP9	BMPR1A	ACVR2A	3.33E-01
	BMP9	BMPR1A	ACVR2B	9.18E+01
	BMP9	BMPR1A	BMPR2	4.46E-03
	BMP10	ACVR1	ACVR2A	2.44E-04
	BMP10	ACVR1	ACVR2B	9.92E-02
	BMP10	ACVR1	BMPR2	4.00E-02
	BMP10	BMPR1A	ACVR2A	1.48E-02
	BMP10	BMPR1A	ACVR2B	6.11E+00
	BMP10	BMPR1A	BMPR2	9.39E-01
	GDF5	ACVR1	ACVR2A	1.00E-04
	GDF5	ACVR1	ACVR2B	1.31E-04
	GDF5	ACVR1	BMPR2	1.00E-04
	GDF5	BMPR1A	ACVR2A	1.00E-04
	GDF5	BMPR1A	ACVR2B	1.19E-03
	GDF5	BMPR1A	BMPR2	1.00E-04
**Efficiency (*ε_{ijk}*)**	BMP4	ACVR1	ACVR2A	1.95E+01
	BMP4	ACVR1	ACVR2B	1.00E-04
	BMP4	ACVR1	BMPR2	4.73E+01
	BMP4	BMPR1A	ACVR2A	1.00E-04
	BMP4	BMPR1A	ACVR2B	3.10E+00

	BMP4	BMPR1A	BMPR2	1.38E-01
	BMP7	ACVR1	ACVR2A	2.54E+01
	BMP7	ACVR1	ACVR2B	6.15E-01
	BMP7	ACVR1	BMPR2	6.43E-01
	BMP7	BMPR1A	ACVR2A	2.83E-01
	BMP7	BMPR1A	ACVR2B	1.00E-04
	BMP7	BMPR1A	BMPR2	4.08E-01
	BMP9	ACVR1	ACVR2A	3.31E+01
	BMP9	ACVR1	ACVR2B	8.89E-02
	BMP9	ACVR1	BMPR2	1.24E+00
	BMP9	BMPR1A	ACVR2A	1.01E-04
	BMP9	BMPR1A	ACVR2B	1.00E-04
	BMP9	BMPR1A	BMPR2	4.81E-03
	BMP10	ACVR1	ACVR2A	5.31E+01
	BMP10	ACVR1	ACVR2B	1.00E-04
	BMP10	ACVR1	BMPR2	1.00E-04
	BMP10	BMPR1A	ACVR2A	1.00E-04
	BMP10	BMPR1A	ACVR2B	1.00E-04
	BMP10	BMPR1A	BMPR2	2.16E-01
	GDF5	ACVR1	ACVR2A	2.58E-02
	GDF5	ACVR1	ACVR2B	1.00E-04
	GDF5	ACVR1	BMPR2	1.00E-04
	GDF5	BMPR1A	ACVR2A	1.00E-04
	GDF5	BMPR1A	ACVR2B	1.00E-04
	GDF5	BMPR1A	BMPR2	2.16E-01
**Receptor rescaling (ρ_j or ρ_k)**	-	ACVR1	-	2.49E+00
	-	BMPR1A	-	2.57E+00
	-	-	ACVR2A	4.50E-01
	-	-	ACVR2B	9.04E-01
	-	-	BMPR2	1.00E+00
